# Constraining remote oxidation capacity with ATom observations

**DOI:** 10.5194/acp-20-7753-2020

**Published:** 2020-07-03

**Authors:** Katherine R. Travis, Colette L. Heald, Hannah M. Allen, Eric C. Apel, Stephen R. Arnold, Donald R. Blake, William H. Brune, Xin Chen, Róisín Commane, John D. Crounse, Bruce C. Daube, Glenn S. Diskin, James W. Elkins, Mathew J. Evans, Samuel R. Hall, Eric J. Hintsa, Rebecca S. Hornbrook, Prasad S. Kasibhatla, Michelle J. Kim, Gan Luo, Kathryn McKain, Dylan B. Millet, Fred L. Moore, Jeffrey Peischl, Thomas B. Ryerson, Tomás Sherwen, Alexander B. Thames, Kirk Ullmann, Xuan Wang, Paul O. Wennberg, Glenn M. Wolfe, Fangqun Yu

**Affiliations:** 1 Department of Civil and Environmental Engineering, Massachusetts Institute of Technology, Cambridge, MA, USA; 2 Department of Earth, Atmospheric and Planetary Sciences, Massachusetts Institute of Technology, Cambridge, MA, USA; 3 Division of Chemistry and Chemical Engineering, California Institute of Technology, Pasadena, CA, USA; 4 Atmospheric Chemistry Observations & Modeling Laboratory, National Center for Atmospheric Research, Boulder, CO, USA; 5 Institute for Climate and Atmospheric Science, School of Earth and Environment, University of Leeds, Leeds, UK; 6 Department of Chemistry, University of California Irvine, Irvine, CA, USA; 7 Department of Meteorology, Pennsylvania State University, University Park, PA, USA; 8 University of Minnesota, Department of Soil, Water and Climate, St. Paul, MN, USA; 9 Dept. of Earth & Environmental Sciences of Lamont-Doherty Earth Observatory and Columbia University, Palisades, NY, USA; 10 Division of Geological and Planetary Sciences, California Institute of Technology, Pasadena, CA, USA; 11 Harvard John A. Paulson School of Engineering and Applied Sciences, Harvard University, Cambridge, MA, USA; 12 NASA Langley Research Center, Hampton, VA, USA; 13 Global Monitoring Division, NOAA Earth System Research Laboratory, Boulder, CO, USA; 14 Wolfson Atmospheric Chemistry Laboratories (WACL), Department of Chemistry, University of York, York, UK; 15 National Centre for Atmospheric Science (NCAS), University of York, York, UK; 16 Cooperative Institute for Research in Environmental Science, University of Colorado, CO, USA; 17 Nicholas School of the Environment, Duke University, Durham, NC, USA; 18 Division of Engineering and Applied Science, California Institute of Technology, Pasadena, CA, USA; 19 Atmospheric Sciences Research Center, University of Albany, Albany, NY, USA; 20 Chemical Sciences Division, NOAA Earth System Research Laboratory, Boulder, CO, USA; 21 School of Energy and Environment, City University of Hong Kong, Hong Kong, China; 22 Atmospheric Chemistry and Dynamics Laboratory, NASA Goddard Space Flight Center, Greenbelt, MD, USA

## Abstract

The global oxidation capacity, defined as the tropospheric mean concentration of the hydroxyl radical (OH), controls the lifetime of reactive trace gases in the atmosphere such as methane and carbon monoxide (CO). Models tend to underestimate the methane lifetime and CO concentrations throughout the troposphere, which is consistent with excessive OH. Approximately half of the oxidation of methane and non-methane volatile organic compounds (VOCs) is thought to occur over the oceans where oxidant chemistry has received little validation due to a lack of observational constraints. We use observations from the first two deployments of the NASA ATom aircraft campaign during July–August 2016 and January–February 2017 to evaluate the oxidation capacity over the remote oceans and its representation by the GEOS-Chem chemical transport model. The model successfully simulates the magnitude and vertical profile of remote OH within the measurement uncertainties. Comparisons against the drivers of OH production (water vapor, ozone, and NO_*y*_ concentrations, ozone photolysis frequencies) also show minimal bias, with the exception of wintertime NO_*y*_. The severe model overestimate of NO_*y*_ during this period may indicate insufficient wet scavenging and/or missing loss on sea-salt aerosols. Large uncertainties in these processes require further study to improve simulated NO_*y*_ partitioning and removal in the troposphere, but preliminary tests suggest that their overall impact could marginally reduce the model bias in tropospheric OH. During the ATom-1 deployment, OH reactivity (OHR) below 3 km is significantly enhanced, and this is not captured by the sum of its measured components (cOHR_obs_) or by the model (cOHR_mod_). This enhancement could suggest missing reactive VOCs but cannot be explained by a comprehensive simulation of both biotic and abiotic ocean sources of VOCs. Additional sources of VOC reactivity in this region are difficult to reconcile with the full suite of ATom measurement constraints. The model generally reproduces the magnitude and seasonality of cOHR_obs_ but underestimates the contribution of oxygenated VOCs, mainly acetaldehyde, which is severely underestimated throughout the troposphere despite its calculated lifetime of less than a day. Missing model acetaldehyde in previous studies was attributed to measurement uncertainties that have been largely resolved. Observations of peroxyacetic acid (PAA) provide new support for remote levels of acetaldehyde. The underestimate in both model acetaldehyde and PAA is present throughout the year in both hemispheres and peaks during Northern Hemisphere summer. The addition of ocean sources of VOCs in the model increases cOHR_mod_ by 3% to 9% and improves model–measurement agreement for acetaldehyde, particularly in winter, but cannot resolve the model summertime bias. Doing so would require 100 Tg yr^−1^ of a long-lived unknown precursor throughout the year with significant additional emissions in the Northern Hemisphere summer. Improving the model bias for remote acetaldehyde and PAA is unlikely to fully resolve previously reported model global biases in OH and methane lifetime, suggesting that future work should examine the sources and sinks of OH over land.

## 1 Introduction

The hydroxyl radical (OH) is the main oxidant responsible for removing trace gases from the atmosphere, and its concentration defines the tropospheric oxidation capacity. OH is primarily produced by the photolysis of ozone in the presence of water vapor. The lifetimes of key atmospheric trace gases are governed by how quickly they are removed by reaction with OH. Oxidation of volatile organic compounds (VOCs) by OH produces tropospheric ozone and fine particulate matter which are detrimental to human health and vegetation and impact climate. The oxidation of VOCs, carbon monoxide (CO), and methane provides the main sink of OH in the troposphere. Oxidation of methane and VOCs accounts for over half of the global CO production (Duncan et al., 2007; Safieddine et al., 2017), resulting in a tight coupling of these compounds.

Models generally overestimate global mean tropospheric OH and its ratio in the Northern Hemisphere to Southern Hemisphere (Naik et al., 2013; Patra et al., 2014). These biases may be linked to the persistent CO underestimate in models (Shindell et al., 2006), as prescribing OH from observations improves simulated CO (Müller et al., 2018). However, constraining models with observations of ozone and water vapor cannot resolve biases in model OH (Strode et al., 2015), which is impacted by additional complex factors such as the chemical mechanism and the ozone photolysis frequency (Nicely et al., 2017). Constraining the performance of model chemical mechanisms has largely focused on regions of strong biogenic and anthropogenic activity (Emmerson and Evans, 2009; Yu et al., 2010; Marvin et al., 2017), but at least half of the oxidation of methane occurs over the ocean, where models have received little evaluation due to a lack of observational constraints.

The introduction of airborne measurements of OH reactivity (OHR) provides a method to evaluate the sink of OH across a range of altitudes and a variety of locations and chemical environments (Mao et al., 2009; Thames et al., 2020). Previous work compared surface observations of OHR at a single site to the sum of individually calculated OHR components from measurements (Di Carlo, 2004; Yoshino et al., 2006; Sinha et al., 2008, 2010; Mao et al., 2010; Dolgorouky et al., 2012; Hansen et al., 2014; Nakashima et al., 2014; Nölscher et al., 2012, 2016; Ramasamy et al., 2016; Zannoni et al., 2016, 2017) or from simple models (Ren et al., 2006; Lee et al., 2009; Lou et al., 2010; Mogensen et al., 2011; Mao et al., 2012; Edwards et al., 2013; Kaiser et al., 2016; Whalley et al., 2016). Thames et al. (2020) found evidence of missing OHR between measurements and an observationally constrained box model during the first three ATom deployments. Chen et al. (2019) compared calculated OHR from a global model to OHR determined from a suite of VOCs but did not have measurements of OHR itself. Ferracci et al. (2018) found that missing OHR estimated from surface observations could result in a small increase in the methane lifetime in a global model. Safieddine et al. (2017) and Lelieveld et al. (2016) presented the first global model simulations of OHR but with only qualitative comparison to observations. No study has quantitatively compared simulated and observed OHR in a global model in an effort to constrain the OH sink.

The ATom campaign (Wofsy et al., 2018) provides an unprecedented opportunity to test models in the remote atmosphere with a detailed suite of chemical observations. We simulate the first two deployments (ATom-1: July–Agust 2016, ATom-2: January–February 2017) using the GEOS-Chem chemical transport model (CTM) as our tool to explore potential sources of systematic errors that could explain the community-wide model overestimate in global mean OH and underestimate of the methane lifetime. We include model evaluation with measurements of OHR, a relatively new constraint available for assessing atmospheric oxidation capacity. To our knowledge, this is the first quantitative use of this measurement to evaluate a CTM.

## 2 Description of model and observations

### 2.1 The GEOS-Chem model

We use the GEOS-Chem global 3-D CTM in v12.3.0 (http://www.geos-chem.org, last access: 2 July 2020) driven by assimilated meteorological data from the Goddard Earth Observing System Modern-Era Retrospective analysis for Research and Applications, Version 2 (MERRA-2; Gelaro et al., 2017). The native MERRA-2 model has a horizontal resolution of 0.5° × 0.625× and 72 vertical levels which we degrade to 2° × 2.5° and 47 vertical levels for use in GEOS-Chem. The midpoint of the first model layer is 58 m. We use time steps of 20 min for chemistry and 10 min for transport as recommended by Philip et al. (2016). GEOS-Chem includes detailed treatment of HO_*x*_–NO_*x*_–VOC–halogen–aerosol chemistry, with recent improvements for isoprene (Chan Miller et al., 2017; Fisher et al., 2016; Marais et al., 2016; Travis et al., 2016), peroxyacetyl nitrate (PAN) (Fischer et al., 2014), and halogen chemistry (Sherwen et al., 2016). The production of organic aerosols is calculated using fixed yields from isoprene, monoterpenes, biomass burning, and anthropogenic fuel combustion (Pai et al., 2020). Aerosol uptake of HO_2_ is parameterized with a reactive uptake coefficient (*γ*) of 0.2 (Jacob, 2000) to produce H_2_O (Mao et al., 2013). Aerosol thermodynamic equilibrium is calculated by ISORROPIA II v2.0 (Pye et al., 2009). Surface methane concentrations are prescribed monthly using spatially interpolated observations from the NOAA GMD flask network. We simulate the 2016–2017 period with an 18-month initialization.

Global fire emissions at 3-hourly resolution (Mu et al., 2011) for 2016 and 2017 are from the Global Fire Emissions Database (GFED4s; van der Werf et al., 2017). The GFED4s burned area (Giglio et al., 2013) includes a parameterization of small fires (Randerson et al., 2012). Biogenic VOC emissions are from MEGANv2.1 (Guenther et al., 2012; Hu et al., 2015). Global anthropogenic emissions are from the Community Emissions Data System (CEDS) inventory (Hoesly et al., 2018), overwritten by ethanol from the POET inventory (Olivier et al., 2003; Granier et al., 2005), ethane from Tzompa-Sosa et al. (2017), and regional inventories for the United States (NEI11v1, Travis et al., 2016), Canada (CAC, https://www.canada.ca/en/services/environment/pollution-waste-management/national-pollutant-release-inventory.html, last access: 31 July 2013), Mexico (BRAVO, Kuhns et al., 2003), Europe (EMEP, http://www.emep.int/index.html, last access: 31 March 2015), Asia (MIX, Li et al., 2017), and Africa (DICE, Marais and Wiedinmyer, 2016). Lightning emissions are constrained with satellite data according to Murray et al. (2012) with a global flash rate of 280 mol NO flash^−1^ (Marais et al., 2018). Air–sea exchange is calculated for acetaldehyde (Millet et al., 2010), acetone (Fischer et al., 2012), and dimethyl sulfide (Breider et al., 2017). All emissions are processed by the Harvard Emissions Component (HEMCO, Keller et al., 2014). Table 1 gives the 2016 emission budget for CO and NO_*x*_.

The standard simulation includes prescribed methanol concentrations. We expand this simulation to include emissions and chemistry for methanol as well as unsaturated C_2_ compounds. Air–sea exchange of methanol is specified using the methodology of Millet et al. (2008) with a constant seawater concentration of 142 nM. Terrestrial biogenic methanol emissions are from MEGANv2.1, and anthropogenic and biomass burning emissions are from the inventories described above. We likewise include biomass burning and anthropogenic emissions of ethyne (C_2_H_2_) and ethene (C_2_H_4_) along with terrestrial biogenic emissions of C_2_H_4_. Oxidation of C_2_H_2_ by OH proceeds according to the Master Chemical Mechanism (MCM) v3.3.1 (Jenkin et al., 1997, 2015; Saunders et al., 2003), via http://mcm.leeds.ac.uk/MCM (last access: 8 February 2018). Simplified C_2_H_4_ chemistry is included based on Lamarque et al. (2012) with an updated OH rate constant from the MCM v3.3.1. Table S1 in the Supplement shows the reactions and species included for unsaturated C_2_ compounds. The standard model does not consider the OH reactivity of a subset of organic acids (RCOOH) from the oxidation of VOCs. We implement oxidation of RCOOH and evaluate the impact of excluding this species, which is minor, in Table S2 and Fig. S1 in the Supplement. The model concentration of H_2_ is fixed at 500 ppt, consistent with observed H_2_ from ATom-1 and ATom-2 (520 ppt).

The GEOS-Chem global mean tropospheric OH ([OH]_GM_) is calculated as an air-mass-weighted quantity below the model tropopause (see http://wiki.seas.harvard.edu/geos-chem/index.php/Mean_OH_concentration, last access: 4 May 2020, for the calculation methodology). The [OH]_GM_ for 2016 is 11.9 × 10^5^ molecules cm^−3^ and the corresponding methane lifetime(*τ*_CH4_) is 9.0 years. This result is comparable to the multi-model [OH]_GM_ of 11.1 × 10^5^ molecules cm^−3^ and *τ*_CH4_ of 9.7 years from Naik et al. (2013). The best observationally derived estimate of *τ*_CH4_ is 11.2 ± 1.3 years (Prather et al., 2012), suggesting a model bias here of 20%. We calculate the ratio of total 2016 air-mass-weighted OH in the Northern (> 0° N) to Southern Hemisphere (< 0° S) to be 1.14. This exceeds observationally derived ratios of 0.85 to 0.97 (Montzka et al., 2000; Patra et al., 2014) but is at the low end of previous model estimates ranging from 1.13 to 1.42 (Naik et al., 2013).

### 2.2 Calculated OH reactivity

The atmosphere contains thousands of reactive organic compounds (Goldstein and Galbally, 2007). Transforming the concentrations of these compounds and reactive inorganics to calculated OH reactivity (cOHR) ranks them in order of their importance as OH sinks. The cOHR from a model (cOHR_mod_) can then be compared to cOHR from a suite of measurements (cOHR_obs_) where cOHR is defined by Eq. (1).

(1) $$\text{cOHR}\left({\text{s}}^{-1}\right)=k_{\text{OH},{\text{CH}}_{4}}\left[{\text{CH}}_{4}\right]+k_{\text{OH},\text{CO}}[\text{CO}]+k_{\text{OH},{\text{NO}}_{2}}\left[{\text{NO}}_{2}\right]+\sum k_{\text{OH},\text{VOC}}[\text{VOC}]+\ldots$$

**Figure 1.**
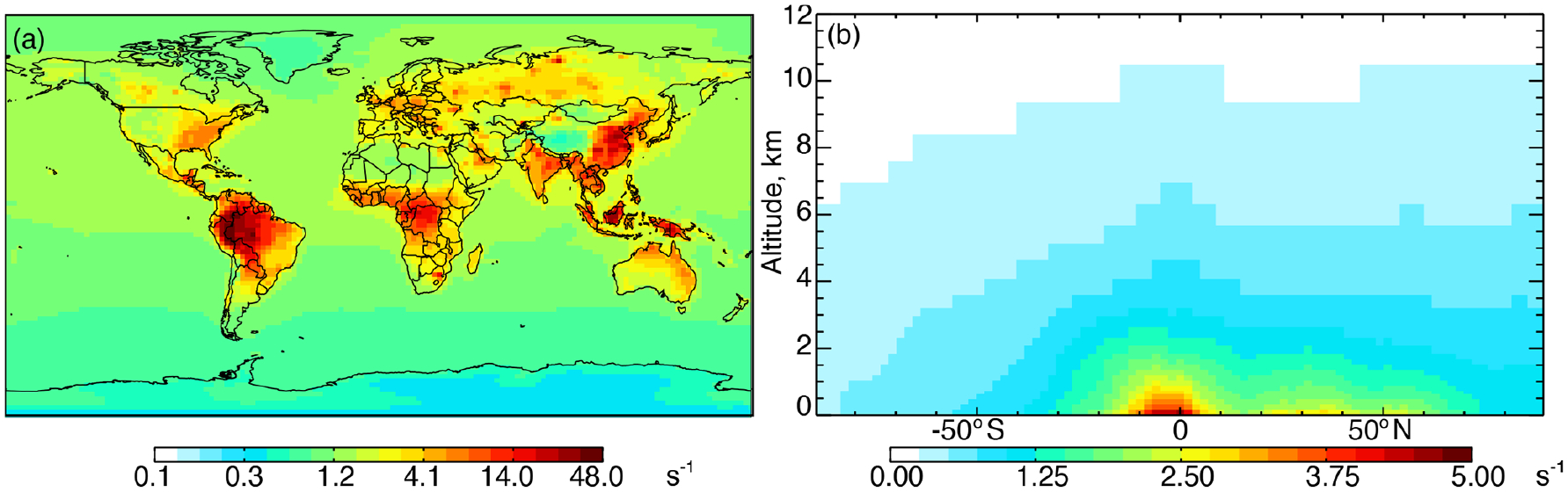
Annual mean 2016 **(a)** surface (log scale) and **(b)** zonal mean cOHR calculated from individual model species. The GEOS-Chem species included in the calculation of cOHR are listed in Table S3.

Figure 1a shows annual surface cOHR_mod_ for the year 2016 based on the 90 components listed in Table S3. Figure 1b shows the zonal mean profile below 12 km. Approximately 80% of air-mass-weighted cOHR_mod_ resides below 3 km. The average annual surface cOHR_mod_ is 1.8 s^−1^, with 40% present over the ocean (average of 1.0 s^−1^). Higher cOHR_mod_ occurs in coastal outflow regions and the lowest cOHR_mod_ is present over the Southern Ocean. The maximum cOHR_mod_ (48 s^−1^) over northern China is due to high concentrations of SO_2_, NO_*x*_, and CO. In the tropics, elevated cOHR_mod_ is mainly from isoprene, other biogenic species, and CO.

### 2.3 ATom observations

**Figure 2.**
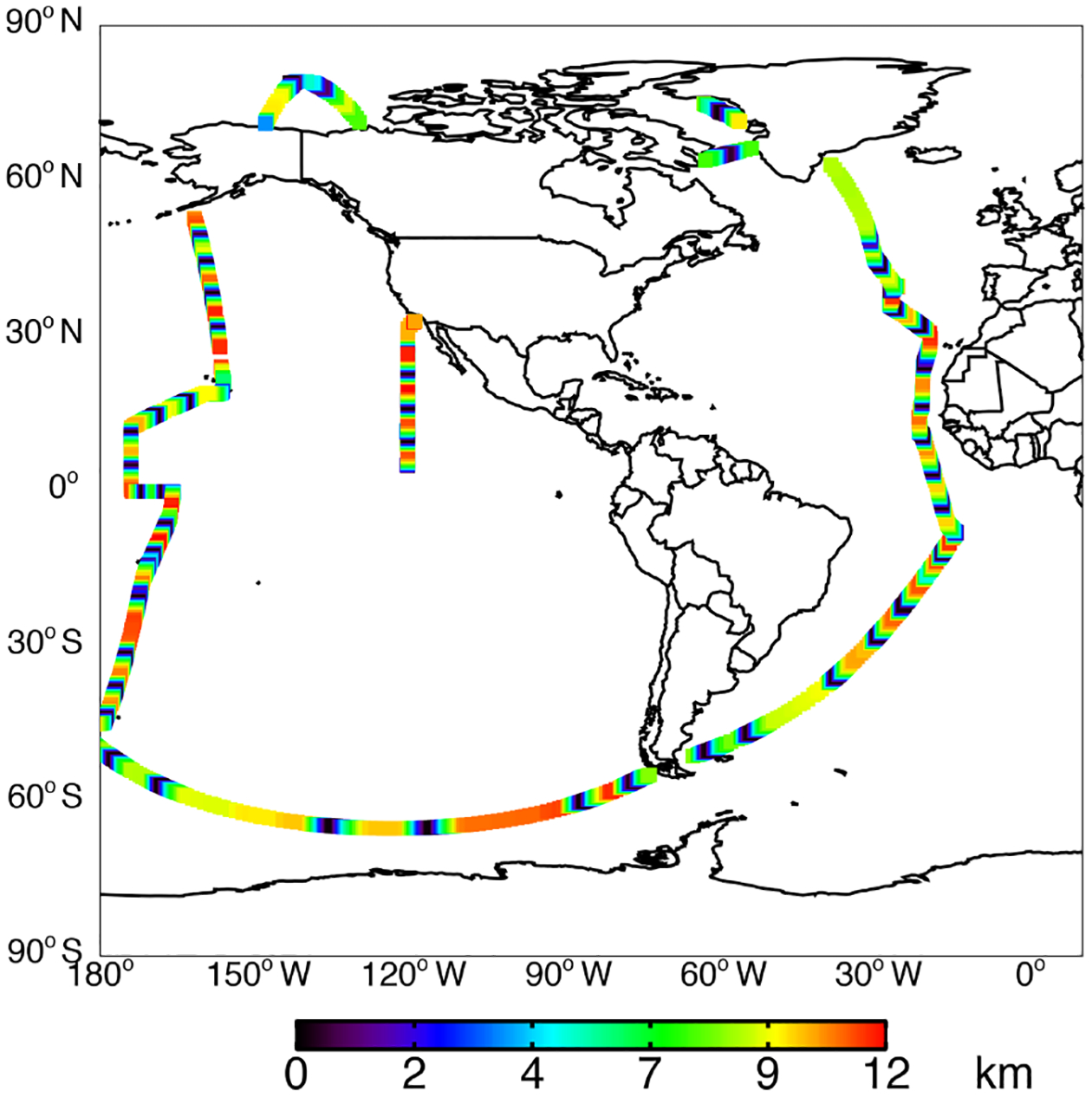
ATom-1 ocean-only flight tracks colored by altitude.

The NASA ATom field campaign (Wofsy et al., 2018) sampled the remote troposphere with the DC-8 aircraft over the Atlantic and Pacific oceans from approximately 200 m to 12 km altitude in four seasons from 2016 to 2018 with a goal of improving the representation of trace gases and short-lived greenhouse gases in models of atmospheric chemistry and climate. We use data here from the first two deployments (ATom-1 and ATom-2), which sampled winter and summer conditions in each hemisphere. We consider only observations over the ocean (73% of measurements). Flight tracks for ATom-1 with land crossings removed are shown in Fig. 2; ATom-2 flight tracks are nearly identical. We sample the model along the flight tracks, and both the model and observations are averaged to the model grid and time step for all the following comparisons. The aircraft carried an extensive chemical payload including observations of water vapor, methane, CO, OH, NO_*x*_, VOCs, photolysis frequencies, and OHR. Table 2 describes the observations used in this work.

## 3 Comparison of simulated and measured OH

**Figure 3.**
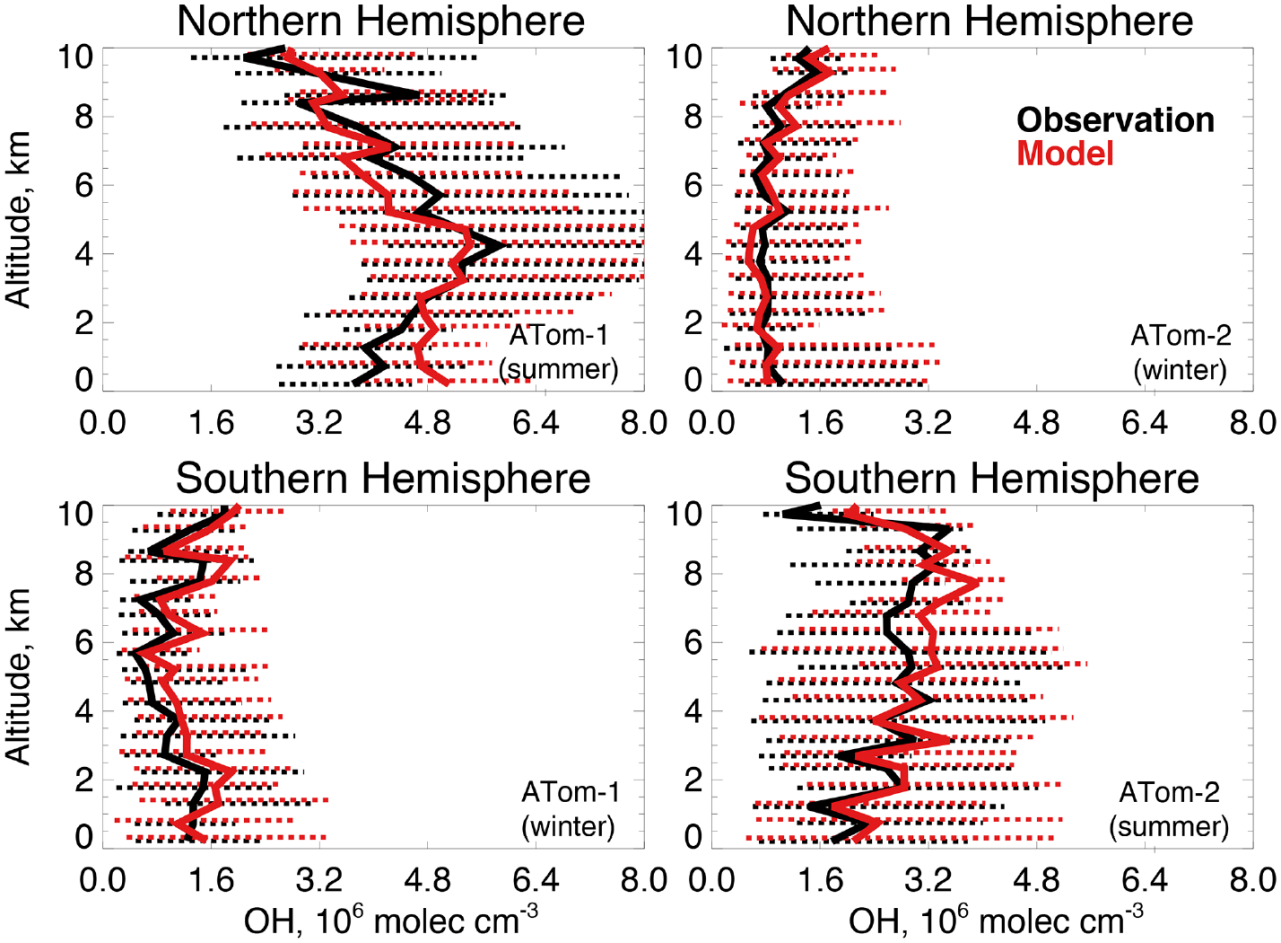
Median OH concentrations for the Northern Hemisphere (> 0° N) and Southern Hemisphere (< 0° S) from the ATHOS instrument described in Table 2 during ATom-1 (July–August 2016) and ATom-2 (January–February 2017) compared against the GEOS-Chem model in 0.5 km altitude bins. The observations have been filtered to remove biomass burning (acetonitrile > 200 ppt) and stratospheric (O_3_*/*CO > 1.25) influence. The dashed lines show the observed 25th–75th percentiles.

We compare observed and simulated OH concentrations to evaluate whether differences are consistent with the bias in *τ*_CH4_ discussed in Sect. 2.1. Figure 3 shows modeled OH sampled along the flight tracks and compared to observed OH (Table 2) for ATom-1 (boreal summer 2016) and ATom-2 (boreal winter 2017) in each hemisphere from the lowest sampled altitude (~ 200 m) to 10 km. There is no evidence of a systematic overestimate in modeled OH throughout the troposphere. Figure S2 shows similarly good agreement across the observed frequency distributions of OH concentration. A model OH overestimate is apparent in the lowest 2 km in the Northern Hemisphere summer that could indicate excessive OH production or an underestimated sink from emissions of ocean VOCs. Global models tend to overestimate OH against constraints from methyl chloroform observations (Shindell et al., 2006; Naik et al., 2013; Nicely et al., 2017), but we find here that tropospheric OH is successfully simulated within observational uncertainty (74% to 135%, 2*σ* confidence level). This result from a global CTM is consistent with good agreement between OH measurements and a box model during NASA’s Pacific Exploratory Mission – Tropics (PEM-Tropic B) campaign in the clean remote Pacific (Tan et al., 2001) and a similar analysis by Brune et al. (2020) for ATom 1 through 4.

We calculate the median air-mass-weighted column average OH (OH_col_) from the median OH concentrations in Fig. 3 and the total tropospheric air mass over the ocean. During ATom-1, the modeled OH_col_ in the Northern (Southern) Hemisphere is 4.5(1.4) × 10^6^ molecules cm^−3^ compared against the observations of 4.4(1.1) × 10^6^ molecules cm^−3^ during ATom-1. Similarly, during ATom-2, OH_col_ is 0.8(2.8) × 10^6^ molecules cm^−3^ in the model and 0.9(2.6) × 10^6^ molecules cm^−3^ in the observations. Median model OH_col_ is within 30% of observations during both deployments, with the smallest bias in the total column during Northern Hemisphere summer when OH is at a maximum. As discussed above, model OH is overestimated in the lowest 2 km during this period, but this bias is minimized in the column average. The observed air-mass-weighted ratio of Northern to Southern Hemisphere OH, calculated in the same manner as described in Sect. 2, is 2.8 during ATom-1 and 0.2 during ATom-2, indicating a strong seasonality that the model largely reproduces (ratios of 2.3 and 0.2). This ratio is less than the ratio of OH_col_ because there is approximately 30% less air mass over the ocean in the Northern Hemisphere ocean than over the Southern Hemisphere. This seasonality is masked by calculations performed on an annual mean basis. The seasonality in this ratio reported by Wolfe et al. (2019) for satellite-derived OH during ATom-1 and ATom-2 is more modest because they calculate a daily average OH that extends to the tropopause, while here, we use largely daytime aircraft observations below 10 km.

The model is in good agreement with OH measurements during ATom, but the uncertainty in the observations is similar to a recent estimate of the GEOS-Chem model uncertainty for OH of 25% to 40% (Christian et al., 2018). In addition, the lifetime of OH is short (seconds) and atmospheric concentrations are highly variable; thus, direct model comparison to measured OH is insufficient to demonstrate model skill in capturing the broader remote oxidation capacity. Agreement between the model and observations could also result from compensating errors in the OH source and sink. We support the model comparison in Fig. 3 with an evaluation of the key factors governing OH production and loss measured by ATom and investigate potential missing sources of VOCs from the ocean during summertime.

## 4 Constraints on the remote source of OH

In the remote troposphere, OH is primarily produced from the photolysis of ozone in the presence of water vapor (Monks, 2005) and is enhanced by nitrogen oxides (NO_*x*_) from lightning and transport from continental sources. Methane, CO, and VOCs provide the main OH sinks (Murray et al., 2014). We compare the model to ATom-1 and ATom-2 observations of the drivers of the tropospheric OH source (water vapor, ozone, ozone photolysis frequency, NO_*x*_) to determine possible broader sources of model bias.

**Figure 4.**
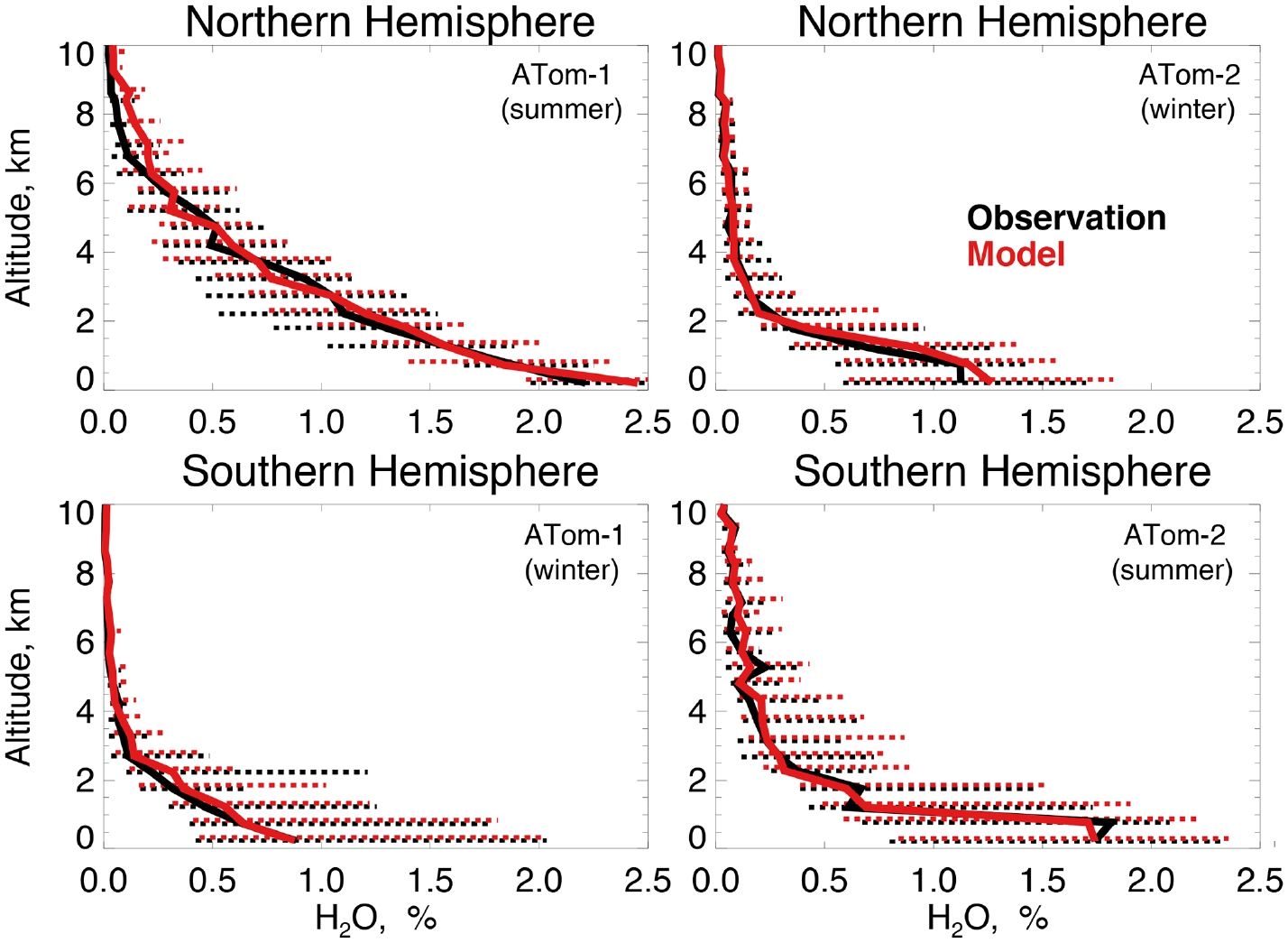
The same as Fig. 3 for median water vapor concentrations. Water vapor mixing ratio was measured by the DLH instrument as described in Table 2.

**Figure 5.**
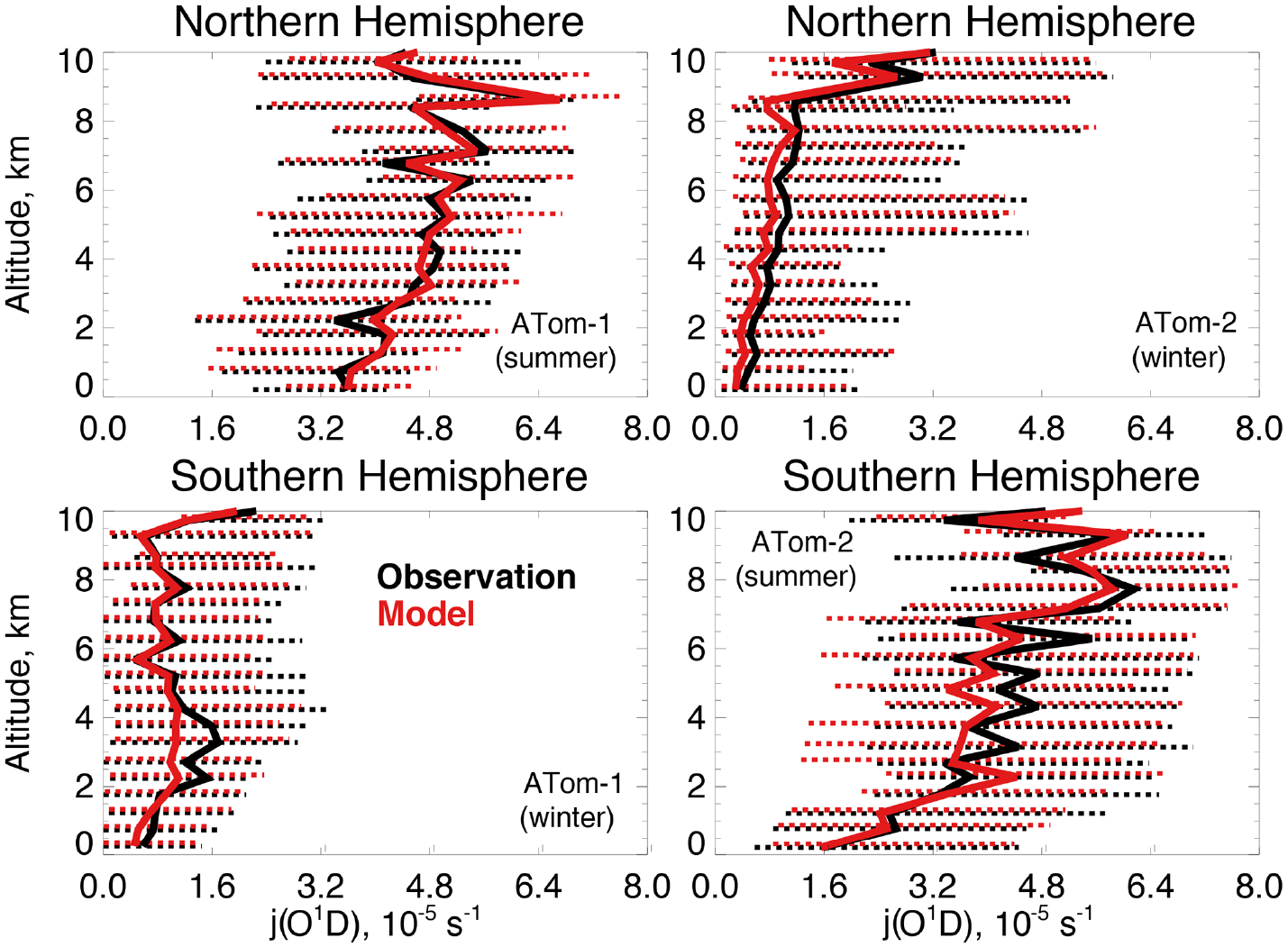
The same as Fig. 3 for median photolysis frequencies for ozone (*j* (O^1^D)). The actinic flux measured by the CAFS instrument is used to calculate *j* (O^1^D) as described in Table 2.

Figure 4 compares observations of water vapor mixing ratios to the NASA MERRA-2 reanalysis product used by the model. MERRA-2 is generally successful at reproducing observed tropospheric water vapor (Gelaro et al., 2017), and we also find good agreement compared with ATom-1 and ATom-2 observations throughout the troposphere. We evaluate the model treatment of the incoming actinic flux and the resulting ozone photolysis frequency (*j* (O^1^D)) in Fig. 5. Hall et al. (2018) showed that GEOS-Chem actinic fluxes in both cloudy and clear skies were well simulated during the ATom-1 deployment. Figure 5 confirms the minimal model bias in *j* (O^1^D) and successful representation of the observed seasonality with median summertime values below 3 km (~ 4 × 10^−5^ s^−1^) approximately 4 times higher than in winter (~ 1 × 10^−5^ s^−1^).

**Figure 6.**
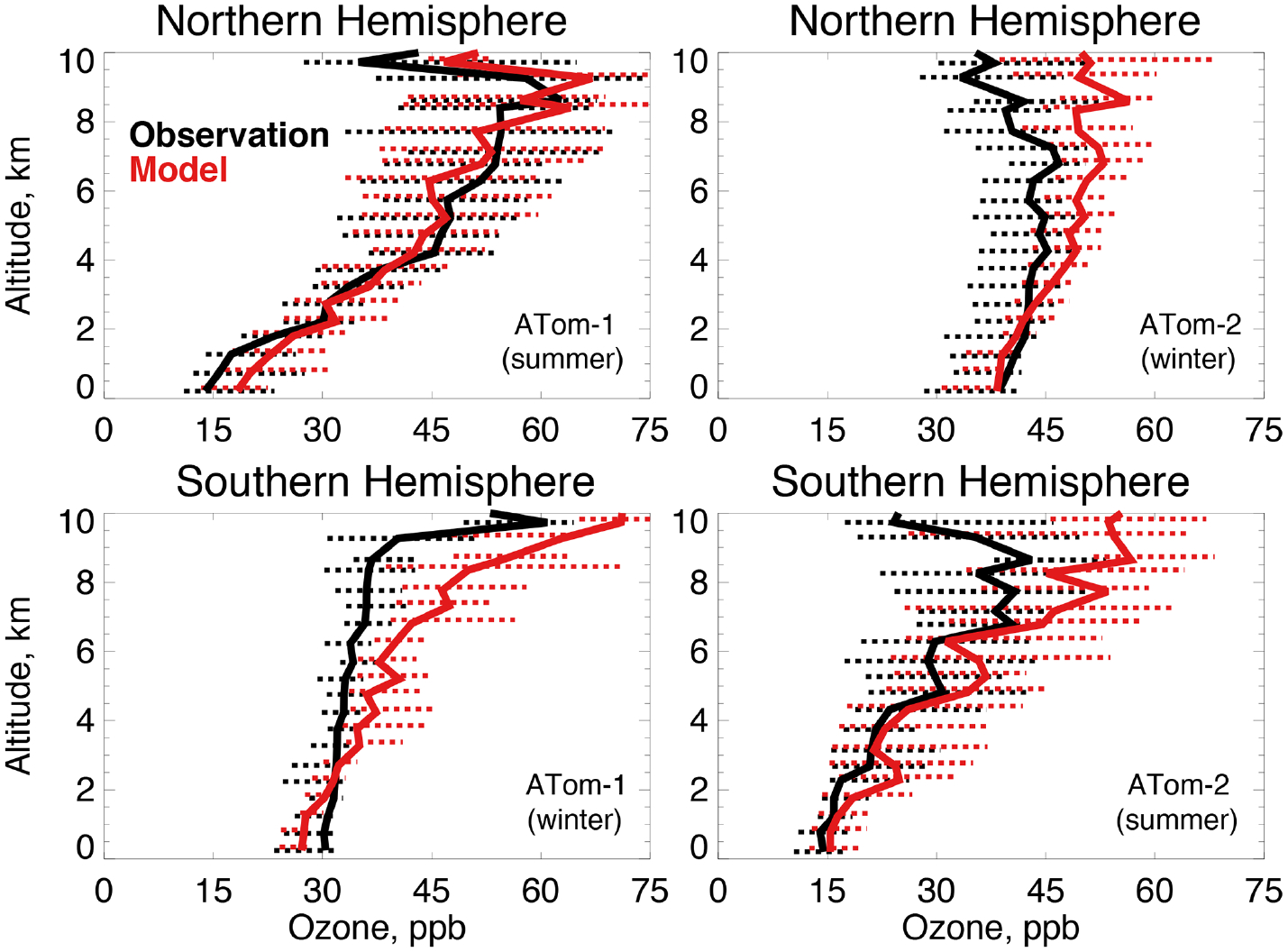
The same as Fig. 3 for median ozone concentrations. Ozone was measured by the NOAA NO_*y*_O_3_ instrument as described in Table 2.

The GEOS-Chem ozone simulation has been extensively tested against ozonesondes, aircraft, and satellite observations and shows no systematic overestimates (Hu et al., 2017), with the exception of continental surface concentrations (Fiore et al., 2009; Travis et al., 2016). Figure 6 shows that the highest (54–63 ppb) and lowest (14 ppb) tropospheric ozone observed during ATom-1 and ATom-2 occurs during summer in the mid to upper troposphere and marine boundary layer, respectively. Ozone is less variable in wintertime, with values between 30 and 50 ppb. The model generally reproduces the magnitude and shape of the tropospheric ozone profiles as well as the seasonality observed during both deployments. There is no evidence of the systematic Northern Hemisphere ozone bias previously seen in global model evaluations (Young et al., 2013) that was suggested as a cause of excessive OH (Naik et al., 2013). This may be reflected in the improved model interhemispheric OH ratio (Sect. 2.1) seen here over previous studies. Upper tropospheric ozone is overestimated in all cases but Northern Hemisphere summer, but this would not have a large influence on primary OH production (or the methane lifetime) at these altitudes (Brune et al., 2020).

**Figure 7.**
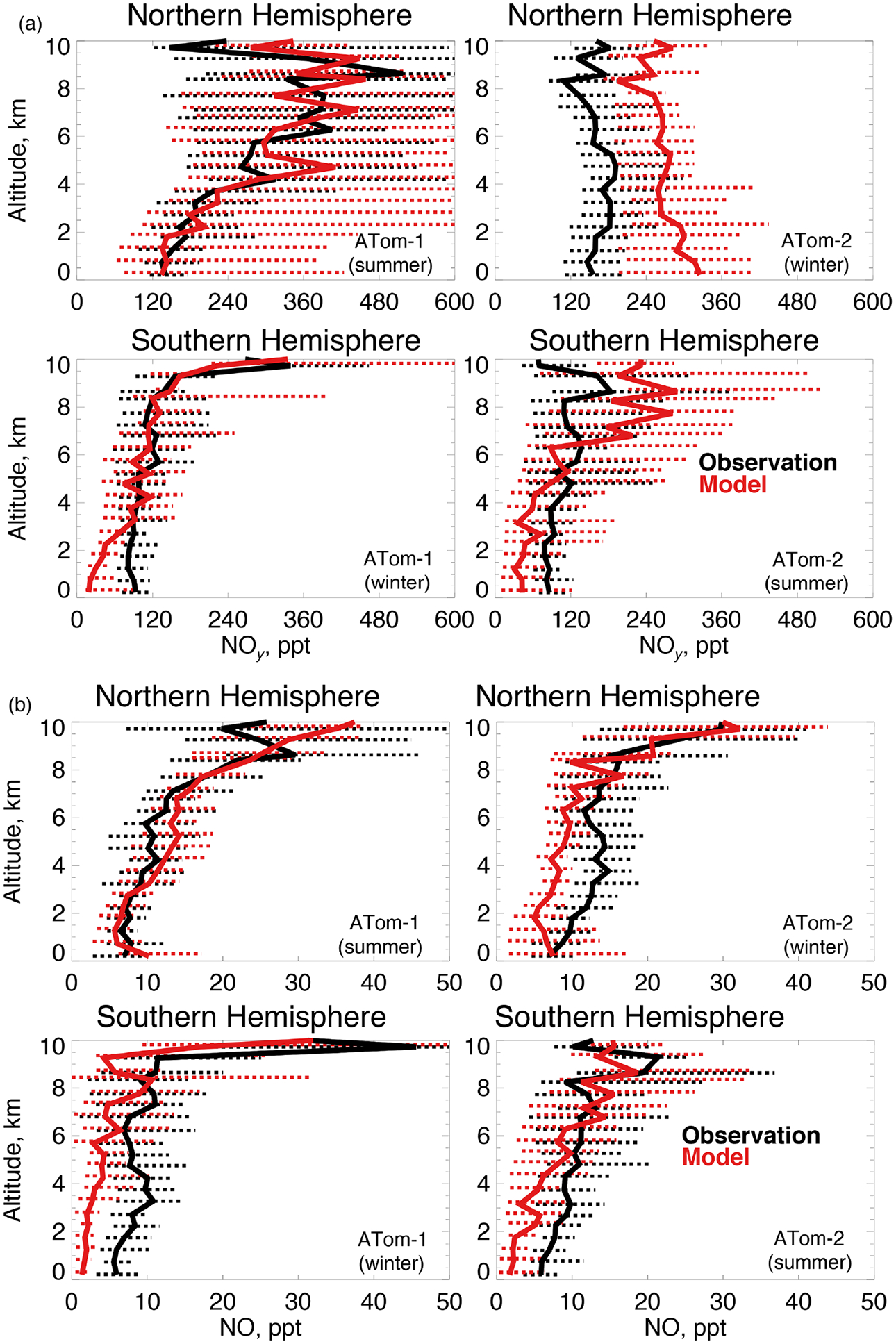
The same as Fig. 3 for median NO_*y*_ **(a)** and NO **(b)** concentrations. NO_*y*_ and NO were measured by the NOAA NO_*y*_O_3_ instrument as described in Table 2.

OH is enhanced in the presence of NO_*x*_ (≡NO + NO_2_). We use NO_*y*_ here (Fig. 7a) as our constraint as observed NO_2_ was generally near the detection limit in both deployments. We also show NO (Fig. 7b) given its role in secondary OH production. The model reproduces the maximum in NO_*y*_ that occurs in the Northern Hemisphere upper troposphere in summertime due to lightning (Marais et al., 2018). Observations show little variability between summer and winter NO_*y*_ in the lower troposphere. Southern Hemisphere NO_*y*_ is underestimated in the lowest few kilometers in both seasons, which could be due to missing ocean production of methyl nitrate (Fisher et al., 2018). The largest model discrepancy is an overestimate of approximately 70% in the Northern Hemisphere wintertime. Observations of NO reflect the structure of NO_*y*_, with the exception of Northern Hemisphere winter.

### Causes of the remote model bias in NO_*y*_

**Figure 8.**
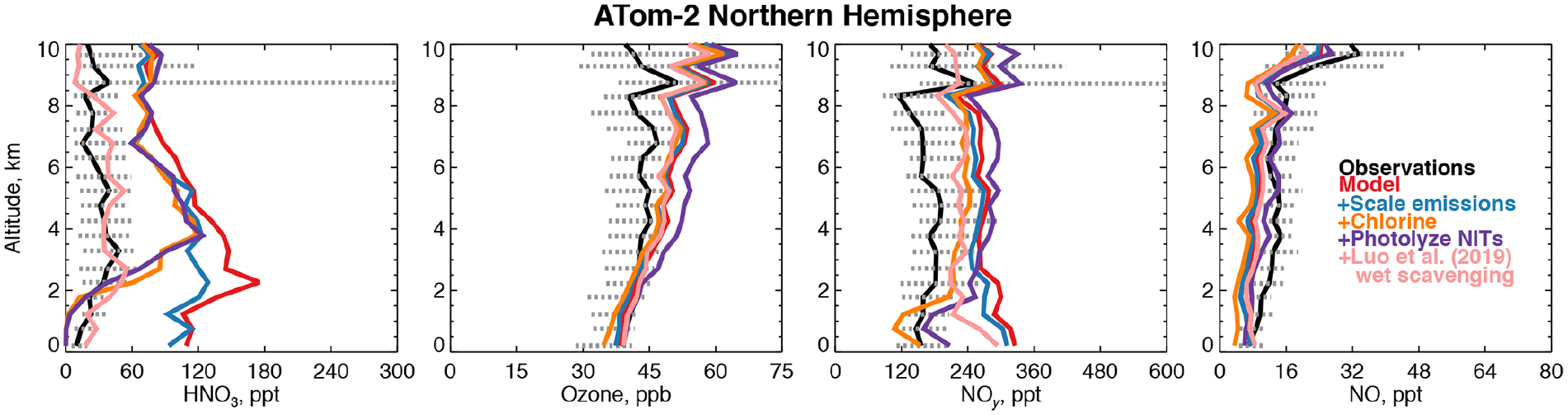
Comparison of modeled and observed HNO_3_, ozone, NO_*y*_, and NO with sensitivity studies including scaling emissions from the US and Asia, improved chlorine chemistry (X. Wang et al., 2019), and the photolysis of particulate nitrate on coarse-mode sea-salt aerosols (Kasibhatla et al., 2018) as described in Sect. 4.1. HNO_3_ was measured by the Caltech CIMS; ozone, NO_*y*_, and NO were measured by the NOAA NO_*y*_O_3_ instrument (Table 2).

Figure 8 shows that the model NO_*y*_ overestimate in winter is primarily caused by nitric acid (HNO_3_). Excessive remote HNO_3_ is a long-standing model deficiency (Bey et al., 2001; Staudt et al., 2003; Brunner et al., 2003, 2005). The model bias identified here is unlikely to result from overestimated continental emissions due to the short lifetime of NO_*y*_ against deposition (~ 3 d in the Northern Hemisphere winter). Models suggest that less than 40% of emitted NO_*x*_ in the US is exported downwind (Dentener et al., 2006; Zhang et al., 2012). However, the standard model configuration here does not address the large possible bias in the US anthropogenic NO_*x*_ inventory of ~ 40% (Anderson et al., 2014; Travis et al., 2016) or the downward trend in NO_*x*_ emissions from Asia of ~ 30% since 2011 (Krotkov et al., 2016). As expected, scaling Asian and US NO_*x*_ emissions by these percentages improves the model bias in winter by only 15% below 3 km (Fig. 8). Recent improvements to the simulation of continental wintertime HNO_3_ (Jaeglé et al., 2018) would similarly be expected to have a marginal effect in our study region.

Kasibhatla et al. (2018) showed that acid displacement of chloride (Cl^−^) by HNO_3_ on sea-salt aerosols (SSA) could resolve model overestimates of gas-phase HNO_3_ in the marine boundary layer using the GEOS-Chem model. A more comprehensive simulation of this process was developed by X. Wang et al. (2019). Figure 8 shows sensitivity tests with the mechanism from X. Wang et al. (2019) incorporated into our simulation in the Northern Hemisphere winter. Model HNO_3_ decreases by approximately 100 ppt below 3 km, which would significantly improve the wintertime NO_*y*_ bias in this region, but the free tropospheric bias remains. The displacement of Cl^−^ described above generates particulate nitrate on coarse-mode SSA (NITs). Photolysis of nitrate has been proposed as a source of NO_*x*_ to the marine boundary layer (Ye et al., 2016; Romer et al., 2018), which might increase HNO_3_. We include NITs photolysis at a frequency of 50 times that of HNO_3_ (Kasibhatla et al., 2018). Figure 8 shows that this mechanism is consistent with observations of NO and ozone below 3 km and does not increase HNO_3_ but increases the free tropospheric NO_*y*_ bias due to PAN formation and exacerbates the overestimate in upper tropospheric ozone during this season.

The difficulty in resolving the bias in wintertime may be due to an overestimate of the NO_*y*_ lifetime as demonstrated by our sensitivities discussed above. Luo et al. (2019) proposed a new treatment of model wet scavenging using spatially and temporally varying cloud condensation water content and an empirical description of HNO_3_ wet removal. This scheme drastically reduced persistent model biases in nitric acid and nitrate at the surface in the United States (Zhang et al., 2012; Heald et al., 2012). As shown in Fig. 8, the revised wet scavenging scheme could fully resolve the remote bias in HNO_3_ throughout the troposphere. However, this parameterization has only received testing over the surface of the continental US, and more evaluation is needed before it can be adopted widely in models.

We find that scaling NO_*x*_, implementing chlorine chemistry, and revised wet scavenging (except in Northern Hemisphere winter) have negative impacts on the modeled OH_col_ along the flight tracks of −1%, −7%, and −4%, respectively. The addition of NITs photolysis to the chlorine chemistry simulation increases OH_col_ by 11% over the base model. In Northern Hemisphere winter only, revised wet scavenging increases OH_col_ by 16%, possibly due to the effect of reduced heterogeneous chemistry. Overall, the annual mean impact of revised wet scavenging from our preliminary sensitivity tests is a −3% reduction in global mean air-mass-weighted OH and a +2% increase in the model methane lifetime. These preliminary sensitivities suggest that resolving the model wintertime NO_*y*_ bias in the Northern Hemisphere could marginally reduce the overestimates of global mean OH on an annual basis if the photolysis frequency of NITs is smaller than 50 times the rate of HNO_3_ photolysis. Recent work from the NASA KORUS-AQ field campaign found that a rate of 1 to 30 might be more consistent with observational constraints (Romer et al., 2018).

Overall, the main drivers of remote tropospheric OH production in our base-case simulation are in good agreement with observations from the first two ATom deployments, with the exception of an NO_*y*_ overestimate in the Northern Hemisphere wintertime. Acid displacement of Cl^−^ by HNO_3_ on SSA (Kasibhatla et al., 2018; X. Wang et al., 2019) may somewhat improve remote HNO_3_ below 3 km, but if the resulting NITs undergoes photolysis at a rate of 50 times that of HNO_3_ (Kasibhatla et al., 2018), the impact on remote NO_*y*_ may be lessened due to the formation of PAN. Both mechanisms require significant further study as tropospheric halogen sources and chemistry and the photolysis frequency of NITs are highly uncertain. A new parameterization of wet scavenging (Luo et al., 2019) would greatly improve modeled remote HNO_3_ and NO_*y*_ but requires further testing and evaluation of its broader impacts on atmospheric chemistry.

## 5 Constraints on the remote sink of OH

The primary sinks of tropospheric OH are CO, methane, and VOCs; OHR measurements represent the sum effect of these species. Previous aircraft measurements of OHR provided evidence of missing reactivity in the remote atmosphere linked to unknown highly reactive VOCs (Mao et al., 2009). During ATom, Thames et al. (2020) measured OHR over the Atlantic and Pacific oceans and determined that missing OHR also correlated with oxygenated VOCs, suggesting the presence of unknown ocean emissions. We compare directly measured OHR during the ATom-1 and ATom-2 deployments to calculated OHR (cOHR_obs_) according to Eq. (1) from the full ATom measurement suite and from the model (cOHR_mod_) sampled along the flight path. Table 2 describes the observations used to calculate cOHR.

**Figure 9.**
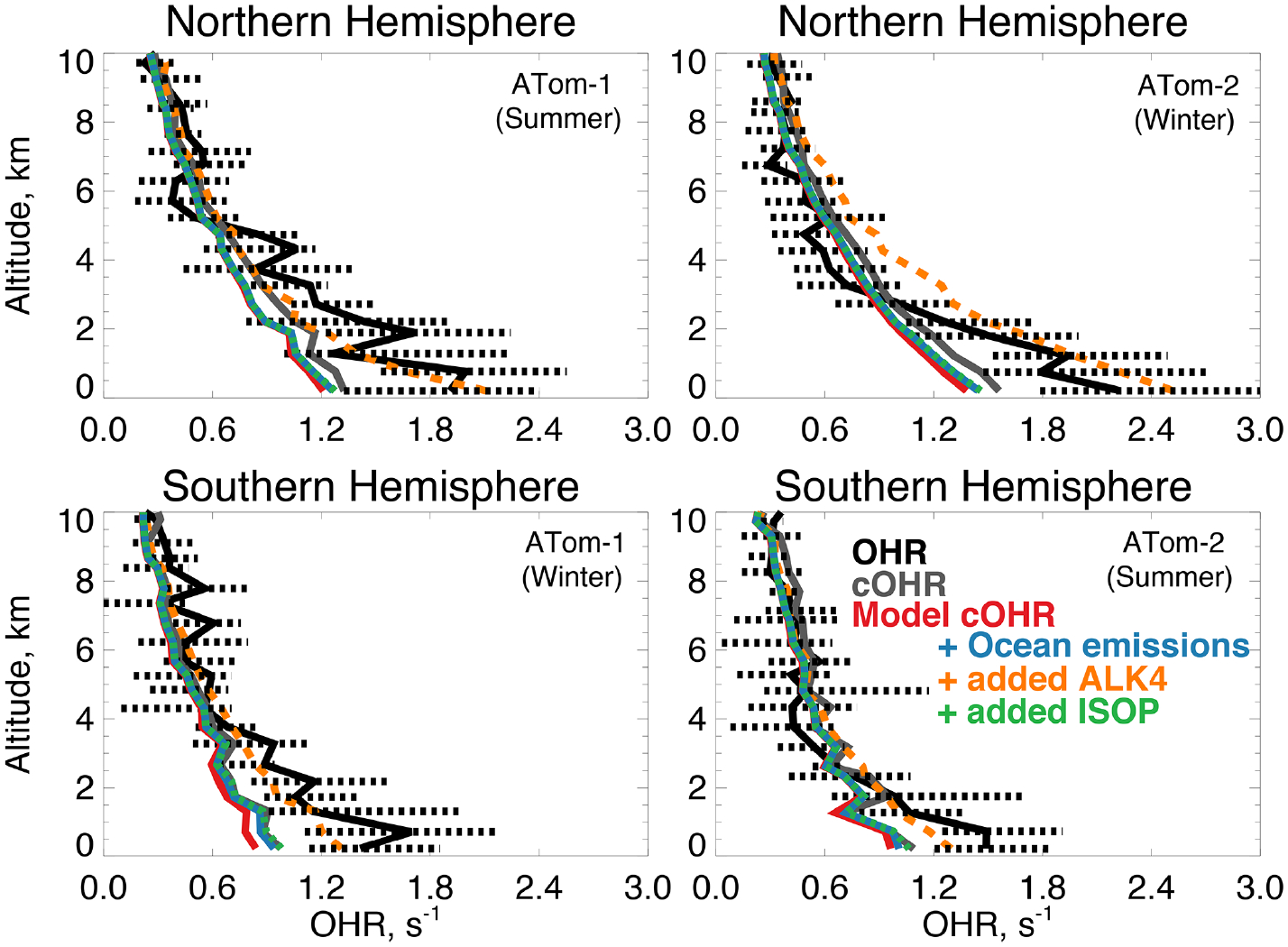
The same as Fig. 3 for median OHR. OHR was measured by the ATHOS instrument as described in Table 2. The calculation of cOHR in the model and observations includes the species described in Table 2. In order to allow for a point-by-point comparison of cOHR in the model and observations, missing values are filled in the observational components of cOHR using linear interpolation. All calculated reactivity values are determined using the temperature and pressure of the ATHOS instrument inlet, which differ from ambient values. The sensitivity tests are described in Sect. 5.

Figure 9 shows the comparison of OHR and cOHR from the model and observations. The observed cOHR is typically less than observed OHR. Along the flight tracks, cOHR_obs_ and cOHR_mod_ show good agreement and strong correlation (*r*^2^ = 0.95 for ATom-1 and ATom-2). The model underestimates cOHR_obs_ by 10% to 12% in the lowest 3 km; we discuss this difference below. The measured relationship between OHR and cOHR_obs_ is weaker (*r*^2^ = 0.53 for ATom-1, *r*^2^ = 0.56 for ATom-2) and cOHR_obs_ is less than OHR below 3 km by 0.2 to 0.4 s^−1^. Thames et al. (2020) showed that median missing reactivity (between OHR and an observationally constrained box model) below 4 km during the ATom-1, ATom-2, and ATom-3 deployments was between 0.2 and 0.8 s^−1^. They provided statistical evidence that while near the level of the instrument accuracy, missing OHR in the marine boundary layer was statistically significant. We find that missing OHR is not associated with acetonitrile or CO (*r*^2^ < 0.06), indicating that biomass burning is not the cause. Acetaldehyde in Northern Hemisphere summer has the strongest relationship with missing OHR (*r*^2^ = 0.19, *p*-value ≪ 0.01, Fig. S3), which suggests a potential role for unmeasured reactive VOCs or their oxidation products from the ocean, as also suggested by Read et al. (2012) and Thames et al. (2020).

Ocean emissions of VOCs have been suggested as a source of remote secondary organic aerosols (Gantt et al., 2010; Kim et al., 2017; Mungall et al., 2017), but their impact on remote reactivity has not been quantified. Our base simulation, described in Sect. 2.1, only includes air–sea exchange of acetone, acetaldehyde, methanol, and dimethyl sulfide. We determine whether additional compounds emitted from the ocean, but not generally included in models, could increase cOHR_mod_ and reconcile the observed discrepancy described above. We follow the standard methodology for air–sea exchange described in Millet et al. (2008) to include emission of the species listed in Table 3 using available measured seawater concentrations, with the addition of isoprene implemented as a direct emission according to Arnold et al. (2009). As shown in Table 3, air–sea exchange represents a net sink of VOCs on an annual basis (−75 Tg C yr^−1^), but this is mainly due to ocean uptake of acetone, which is a negligible component of cOHR.

Interfacial photochemistry may provide an additional abiotic source of VOCs from the ocean. We model abiotic ocean emissions of VOCs according to Brüggemann et al. (2018) by applying species-specific emission factors to the monthly ocean photochemical potential derived in their study. We use the emission factor appropriate for the upper bound of this source according to Brüggemann et al. (2017) (Table S4). Table 4 provides a breakdown of these additional VOCs with a total annual emission of 28 Tg C yr^−1^.

**Figure 10.**
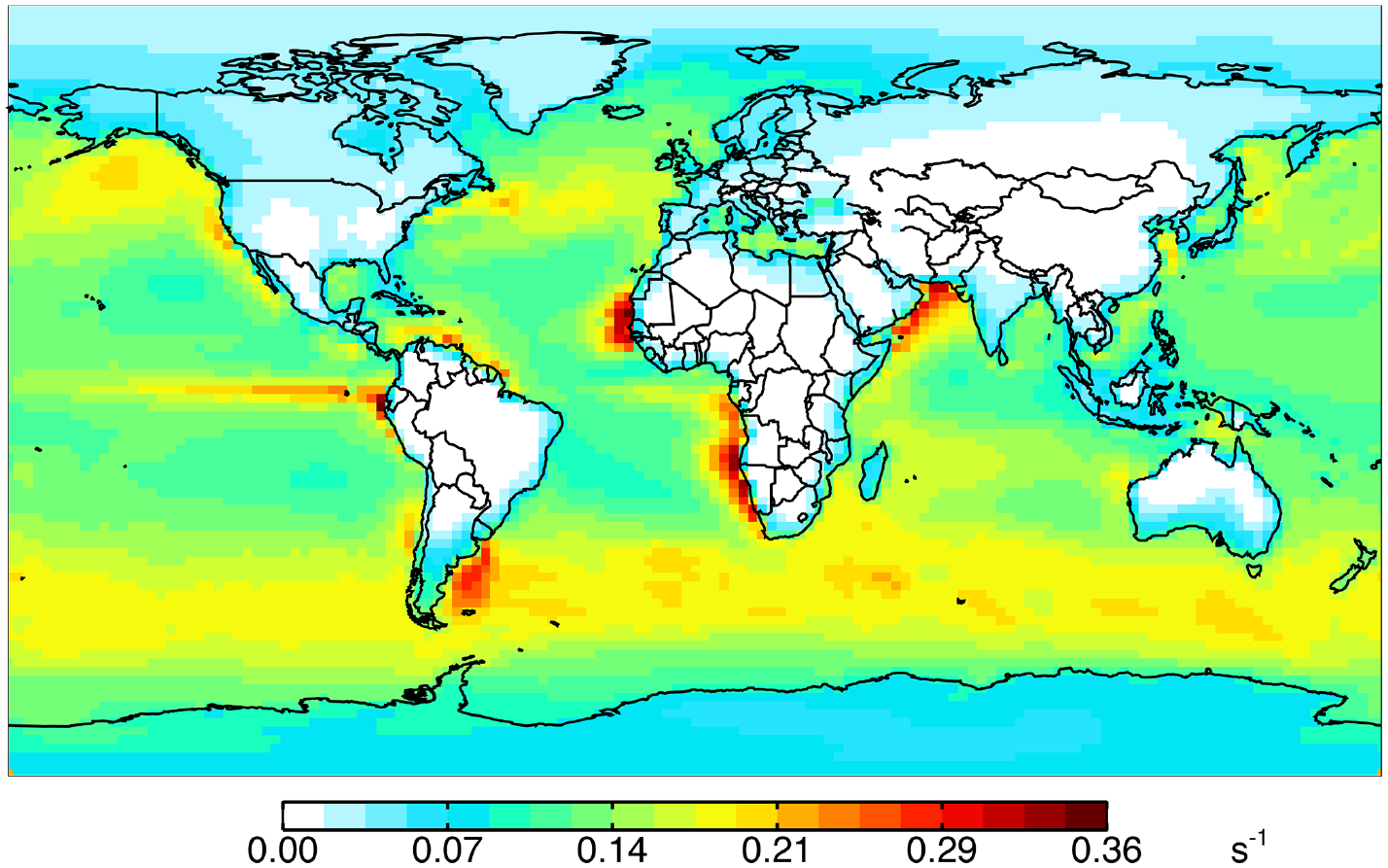
Impact of all ocean emissions (Tables 3 and 4) on annual simulated 2016 surface cOHR as described in the text.

Figure 10 shows the annual mean impact of all ocean emissions described in Tables 3 and 4 (including an adjustment to the acetaldehyde seawater concentration described below in Sect. 5.1) on cOHR_mod_ by turning off those ocean sources in a 1-year simulation. Average annual surface cOHR_mod_ over the ocean increases by 6% over the base simulation and 12% over the simulation with no ocean emissions. The largest increases occur in regions of higher biogenic activity along coastlines and in the Southern Ocean due to the adjustment to acetaldehyde emissions discussed in Sect. 5.1. The incremental impact of the additional ocean emissions over the base simulation is shown in Fig. S4. Without any ocean emissions, global mean OH would be 2% greater than in the case with comprehensive treatment of ocean VOCs. Figure 9 shows that along the flight tracks, cOHR_mod_ increases below 3 km by 3% to 9%, which reduces the model bias against cOHR_obs_. However, the majority of the added species (Tables 3 and 4) were measured during ATom, would therefore contribute to cOHR_obs_, and cannot explain the gap in OHR.

We evaluate the impact of further expanding the oceanic source of reactive VOCs to reconcile the discrepancy between cOHR_obs_ and OHR in a similar manner to Mao et al. (2009). Here, we test a source of alkanes as previously suggested by Read et al. (2012), using the model species ALK4 (> C_4_ alkanes) that has a calculated lifetime of less than 2 d in the Northern Hemisphere summer (*k*_OH_ 2.3 × 10^−12^ cm^3^ molecules^−1^ s^−1^ at 298 K). Known alkanes have been measured in seawater (Plass-Dülmer et al., 1993), but the implied source is small. Consequently, we use ALK4 for testing only. Generating the missing OHR in this way requires an implausibly large oceanic ALK4 source of approximately 340 Tg C yr^−1^ compared against all other sources of VOCs in the model (Tables 3 and 4). A sensitivity test with this source, shown in Fig. 9, largely closes the gap between cOHR_mod_ and OHR but would result in a 20% to 50% reduction in OH below 3 km, biasing the model OH simulation (Fig. 3) and degrading model NO_*y*_ (Fig. 7) due to increased PAN formation.

Thames et al. (2020) found that a partial recycling of OH would be required to maintain consistency with observed OH and HO_2_ during ATom when adding an unknown source of reactivity. If the unknown VOC we suggest includes some OH recycling in its oxidation mechanism and does not produce PAN, the model bias in OH could be mitigated. We use isoprene as our test of a more reactive VOC that includes OH recycling by scaling the ALK4 emission source by the reaction rate of isoprene with OH to obtain a more reasonable emission source of approximately 9 Tg C yr^−1^. Figure 9 shows that this source actually has a minimal impact on cOHR_mod_ of no more than 0.1 s^−1^. Only one-third in summer and two-thirds in winter of the additional cOHR_mod_ from the ocean source of ALK4 are attributable to ALK4; the rest is due to CO, acetaldehyde, and other aldehydes from both increased chemical production and longer lifetimes from suppressed OH. Therefore, a larger source of even a reactive VOC like isoprene is required to close the gap in missing OHR. Reconciling cOHR_mod_ and OHR is therefore difficult using the existing suite of ATom measurement constraints and possible known precursors; further investigation of the accuracy of the OHR measurements in challenging remote conditions may be needed.

**Figure 11.**
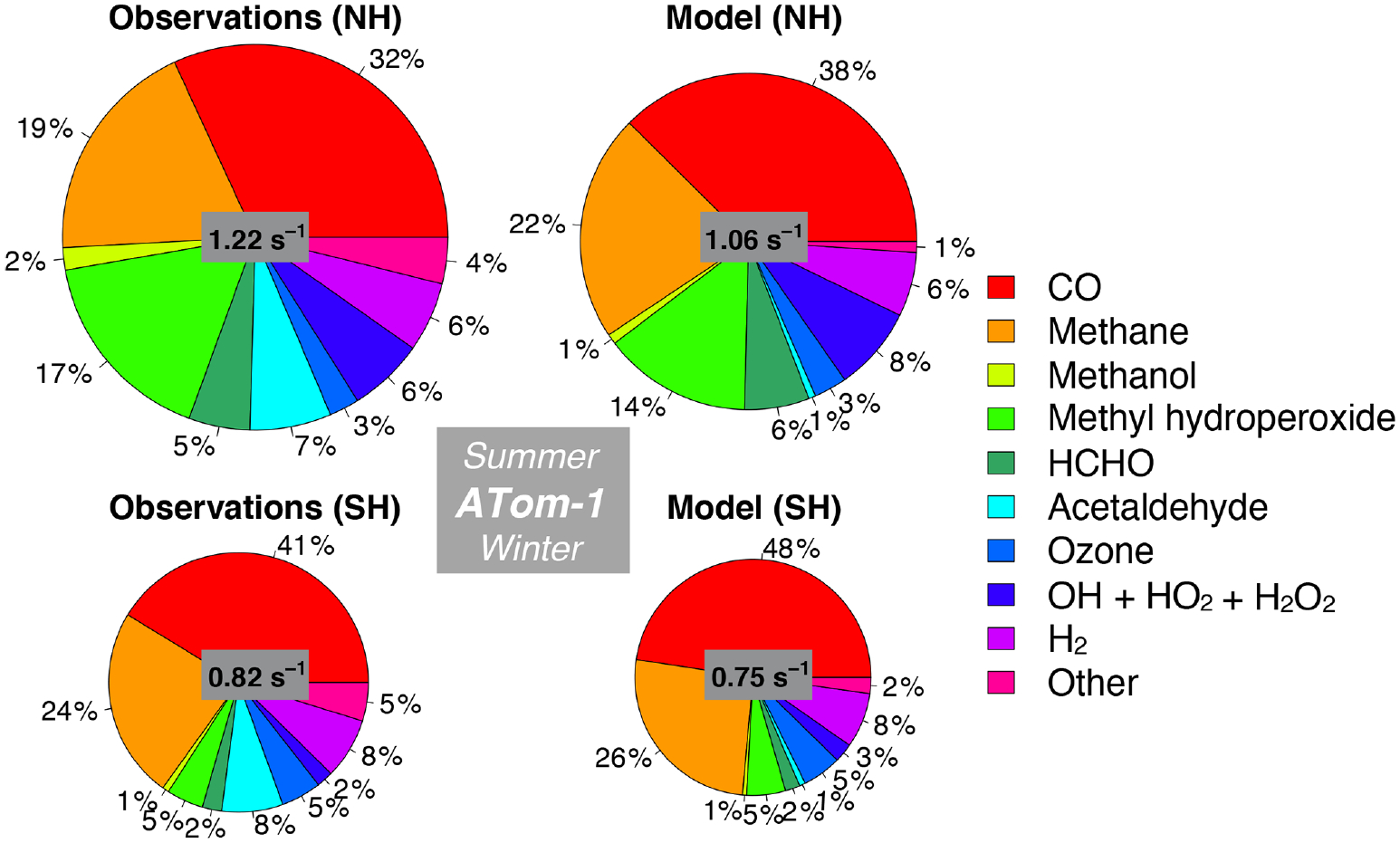
Median observed and modeled OHR and cOHR (see text) below 3 km in the Northern Hemisphere (> 0° N) and Southern Hemisphere (< 0° S) during ATom-1. The “Other” category is the following species as described in Table 2: ethanol, propane, ethane, acetone, > C_3_ aldehydes, methyl ethyl ketone, methyl vinyl ketone, methacrolein, benzene, toluene, > C_4_ alkanes, peroxyacetic acid, peroxynitric acid, dimethyl sulfide, nitric acid, NO, and NO_2_. The diameter of each pie chart is scaled relative to the maximum cOHR for ATom-1.

**Figure 12.**
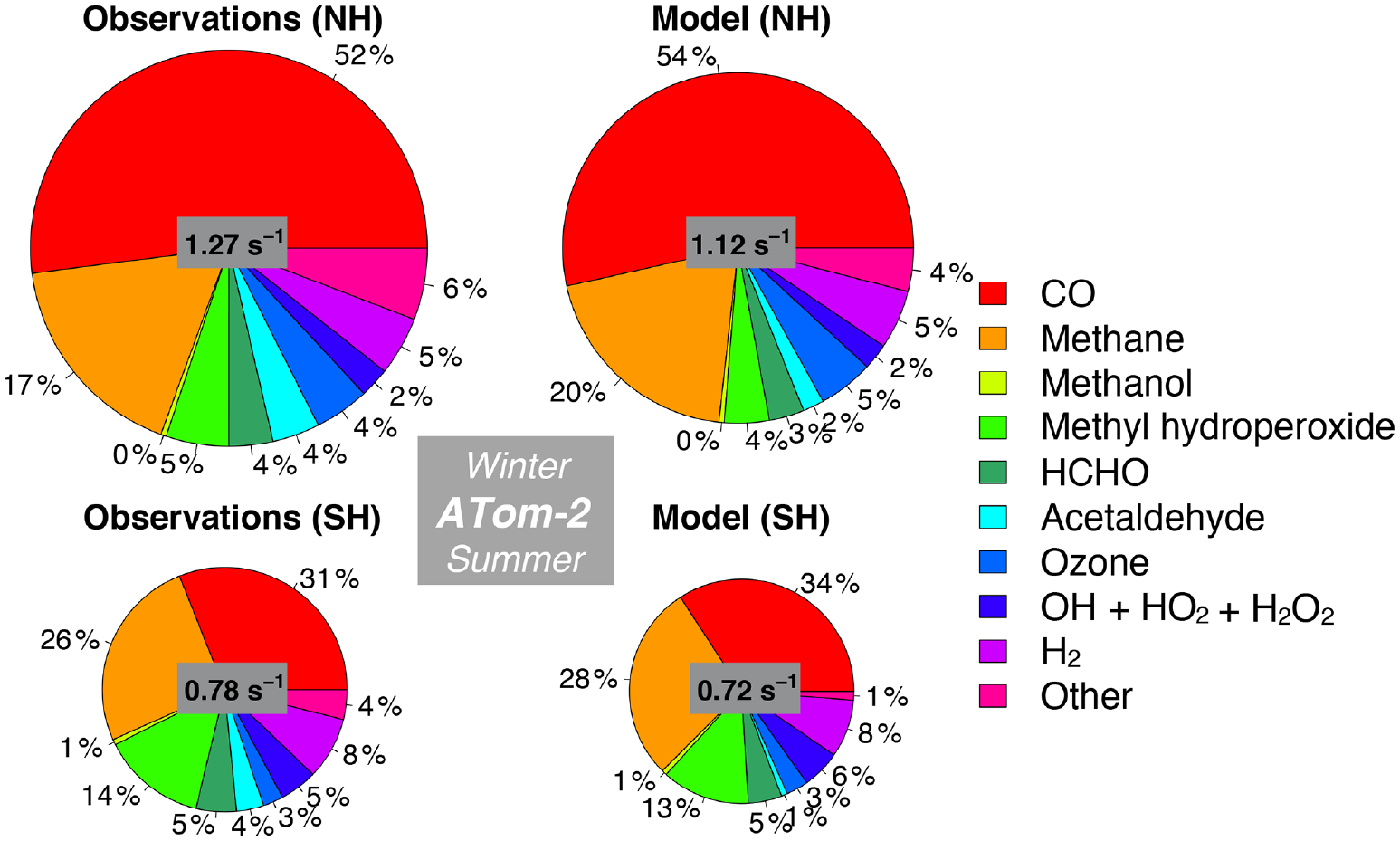
Same as Fig. 10 but for ATom-2. The diameter of each pie chart is scaled relative to the maximum cOHR for ATom-2.

We also assess whether the model accurately represents the components of cOHR_obs_ and explore potential additional sources of missing cOHR_mod_. Figures 11 and 12 show the components of median cOHR in the base simulation below 3 km for each deployment. The composition of cOHR_mod_ is generally consistent with cOHR_obs_. CO and methane make up half or greater of both cOHR_obs_ and cOHR_mod_. There is no systematic underestimate in CO reactivity as might be expected from the general model bias described by Shindell et al. (2006), with the exception of a 9% underestimate during Northern Hemisphere winter when the lifetime of CO is longer and biases in continental sources could have a larger impact. During the ATom-1 deployment, cOHR_obs_ is 50% higher in the Northern Hemisphere (summer) than in the Southern Hemisphere (winter) primarily due to the increase in methyl hydroperoxide (MHP) concentrations. During the ATom-2 deployment, cOHR_obs_ is 60% higher in the Northern Hemisphere (winter) than in the Southern Hemisphere (summer) due to the large contribution of CO in Northern Hemisphere wintertime. The model successfully represents the observed seasonality during both deployments but underestimates cOHR_obs_ by 12% in the Northern Hemisphere and 9% in the Southern Hemisphere.

The difference between measured and simulated cOHR is mainly due to differences between measured and simulated OVOCs. These compounds contribute on average 25% to cOHR_obs_ but 17% to cOHR_mod_. The largest difference in reactivity is from acetaldehyde. Differences between simulated and measured MHP (Fig. S5) are also important and could reflect an error in the MHP lifetime (Müller et al., 2016). However, interferences in the MHP measurement in the boundary layer (Supplement, Sect. S6) have yet to be resolved, and therefore we do not further evaluate causes of underestimated MHP here. We do consider potential missing sources of model acetaldehyde constrained by the ATom measurements over the ocean and assess their impact on simulated OH and CO in Sect. 5.1.

### Evaluation of the remote sources of acetaldehyde

Inability to reconcile remote acetaldehyde observations with models is a long-standing problem (Singh et al., 2001., 2003; Millet et al., 2010; Nicely et al., 2016). Singh et al. (2001) proposed that a large, diffuse, and as-yet unknown source of OVOCs such as acetaldehyde must exist in the troposphere to solve this discrepancy. Read et al. (2012) determined that missing cOHR_mod_ from OVOCs (mainly acetaldehyde) in the marine tropical atmosphere, possibly from terrestrial or ocean sources of alkanes, could cause up to an 8% underestimation of the methane lifetime. Nicely et al. (2016) showed that constraining a box model with observed acetaldehyde reduced tropospheric column OH by 9% and that this acetaldehyde bias was present across eight different CTMs. Therefore, understanding the source of missing acetaldehyde may be part of the cause of the multi-model bias in the methane lifetime and global mean OH.

**Figure 13.**
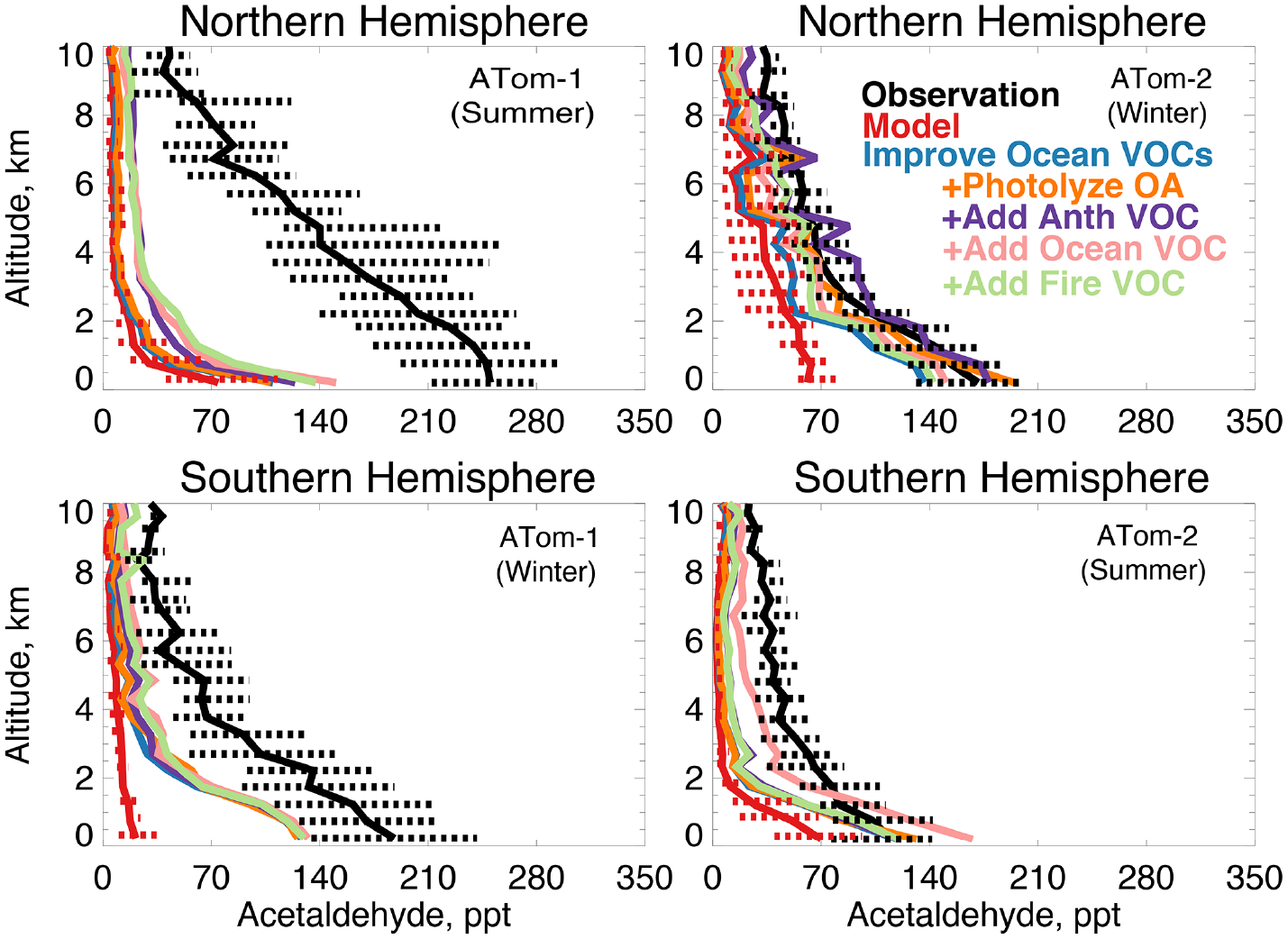
The same as Fig. 3 for median acetaldehyde profiles. Acetaldehyde was measured by the TOGA instrument as described in Table 2. The sensitivity studies are described in Sect. 5.1.

Figure 13 compares the model simulation of acetaldehyde against observations. Average observed concentrations peak in the Northern Hemisphere during ATom-1 with an average mixing ratio of 230 ppt below 3 km and 100 ppt above 3 km despite a lifetime of only several hours in summer. The maximum model underestimate occurs during this period. Observed concentrations are at a minimum during the ATom-2 deployment, indicating a strong seasonality in the source. In each deployment, concentrations remain as high as 70 to 100 ppt as far south as 60° S (Fig. S6), which the model does not reproduce. There is no apparent difference in model bias between observations over the Atlantic or Pacific Ocean (Fig. S7). The model underestimates acetaldehyde on average by 60 to 90% (50 to 200 ppt) below 3 km and does not capture the observed elevated levels throughout the troposphere.

**Figure 14.**
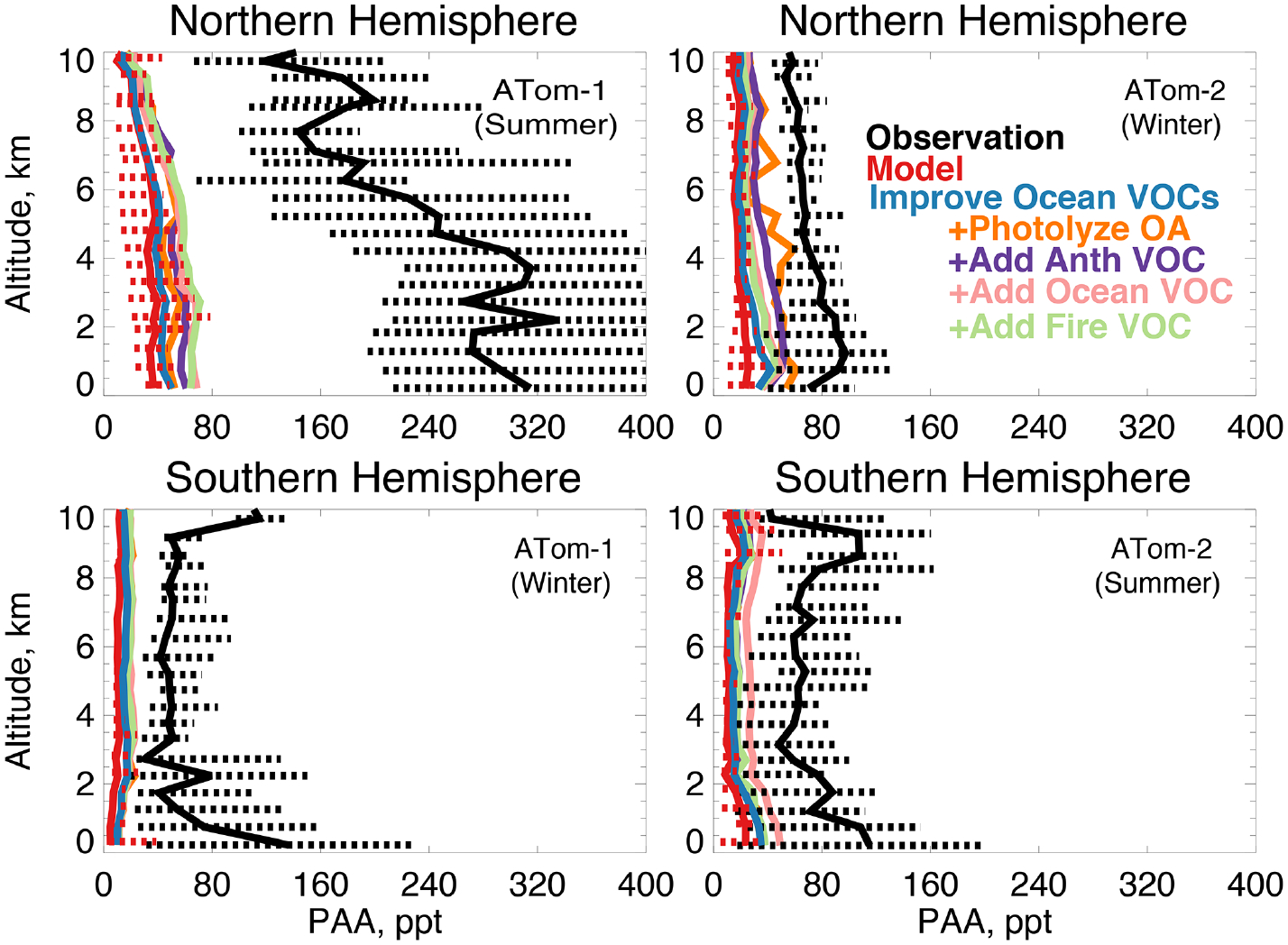
The same as Fig. 3 for median peroxyacetic acid (PAA) profiles. PAA was measured by the Caltech CIMS instrument as described in Table 2. The sensitivity studies are described in Sect. 5.1.

**Figure 15.**
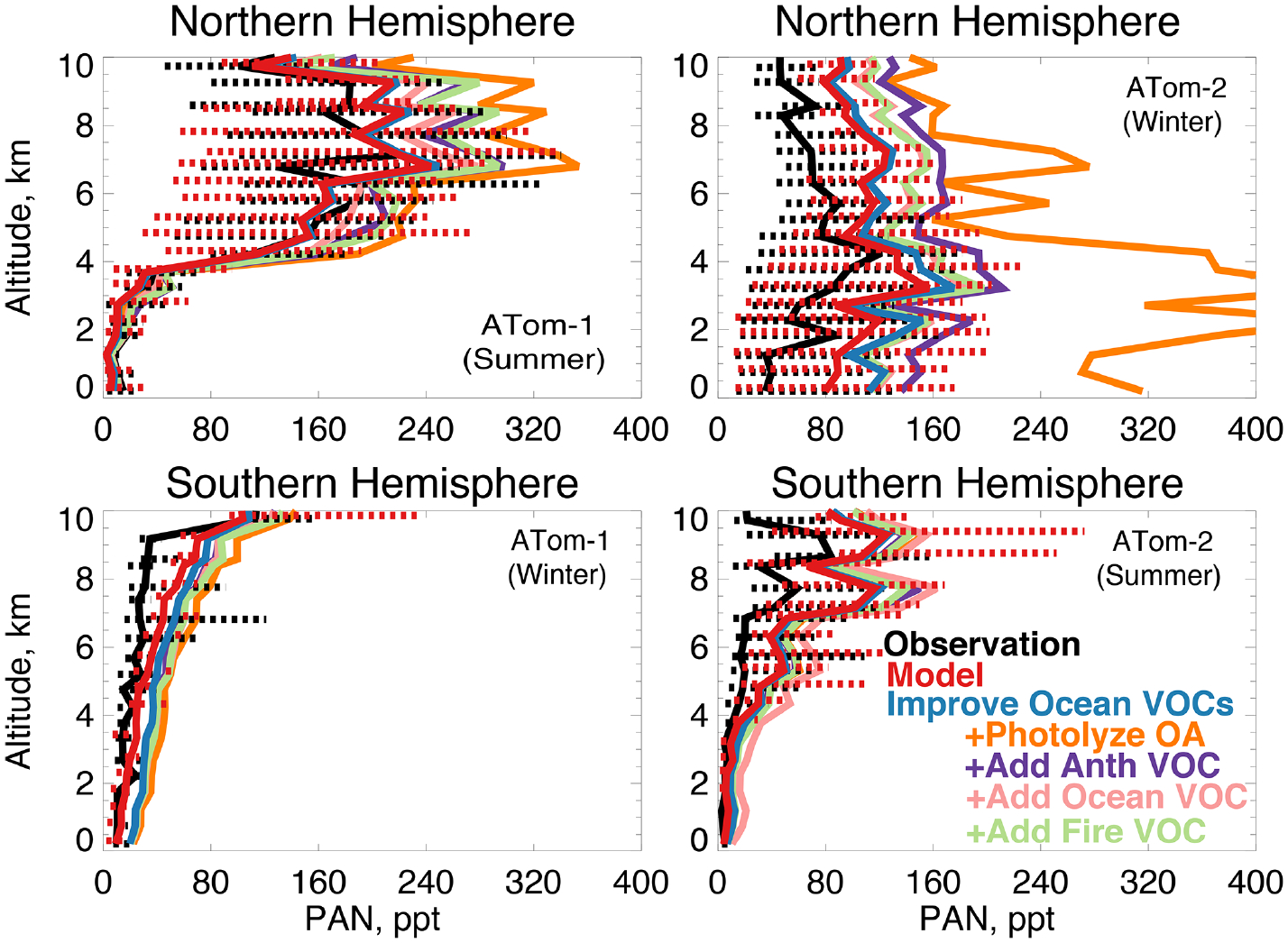
The same as Fig. 3 for median peroxyacetyl nitrate (PAN) profiles. PAN was measured by the PANTHER instrument as described in Table 2. The sensitivity studies are described in Sect. 5.1.

In earlier studies, measurement uncertainties prevented interpretation of model–measurement disagreements in the remote atmosphere, including difficulties in background subtraction (Apel et al., 2008), with uncertainties as high as 70 ppt (Apel et al., 2003), which hindered analysis of clean conditions. The ATom measurement uncertainty is reduced to 10 ppt/20% (Table 2) and does not have the biases present in previous campaigns (S. Wang et al., 2019). Studies have also disputed whether observed acetaldehyde was compatible with observed PAN due to the significant role of acetaldehyde as a PAN precursor through production of the peroxyacetyl (PA) radical (Singh et al., 2001, 2003; Millet et al., 2010). Global simulations estimate that acetaldehyde is responsible for approximately 40% of PA radical production (Fischer et al., 2014), which would be even larger if acetaldehyde is severely underestimated by models. Reaction of the PA radical with HO_2_ is more prevalent in remote environments and produces peroxyacetic acid (PAA) preferentially over PAN, making PAA a more useful constraint for the conditions sampled by ATom. Figure 14 shows the average model underestimate of PAA below 3 km of 70% to 90% (60 to 250 ppt). The model biases for PAA and acetaldehyde both peak with similar magnitude during Northern Hemisphere summer. Figure 15 shows the model comparison with PAN, which is generally well simulated during this period.

S. Wang et al. (2019) used an observationally constrained box model to show that the levels of acetaldehyde observed during ATom are required to explain the observed PAA. The reaction rate of PAA + OH may be 3 times larger (Wu et al., 2017) than the maximum value used by S. Wang et al. (2019), which could result in even better agreement between PAA and acetaldehyde in the marine boundary layer. We evaluate the standard GEOS-Chem acetaldehyde budget, described in detail by Millet et al. (2010), against available ATom observations. The 2016 model budget for the base simulation is provided in Table 5. Acetaldehyde is produced from oxidation of VOCs (ethane, propane, ≥ C_4_ alkanes, ≥ C_3_ alkenes, isoprene, ethanol) and is directly emitted from the ocean, terrestrial plant growth, biomass burning, and anthropogenic activities. The model parameterization of acetaldehyde ocean emissions is dependent on satellite-based observations of colored dissolved organic matter (CDOM) (Millet et al., 2010).

The model free tropospheric bias suggests that long-lived oxidation of VOCs must be underestimated due to the short lifetime of acetaldehyde (< 1 d). The longest-lived precursor VOCs in the model are ethane (2 months) and propane (2 weeks). Ethane has the highest concentration of any measured non-methane VOC during ATom, with an average of 1.5 ppb below 3 km during the Northern Hemisphere winter. The model underestimates average ethane and propane below 10 km by approximately 25% and 60%, respectively (Figs. S8 and S9), which could be due to underestimated natural geologic and fossil fuel emissions (Dalsøren et al., 2018). However, the oxidation of these species is too slow to provide the missing model acetaldehyde and would only marginally increase remote background levels even if it was produced at higher yield at low NO_*x*_ (model yields are ~ 50% for ethane and ~ 20% for propane, Millet et al., 2010). The chemical mechanism used for these species is provided in Table S5. One or more precursors able to resolve the model acetaldehyde bias must therefore be present at higher cumulative concentrations than ethane or propane. Modeled ALK4, parameterized as a butane–pentane mixture, maintains a high acetaldehyde yield at low NO_*x*_ and has a shorter lifetime (~ 5 d), contributing to a larger perturbation to atmospheric acetaldehyde levels than ethane or propane for a given concentration change. The sensitivity test adding substantial ALK4 emissions from the ocean described in Sect. 4 would not resolve the free tropospheric bias in the Northern Hemisphere but would result in a 40% overestimate below 1 km. Furthermore, ALK4 is too short-lived to substantially perturb the remote atmosphere from a continental source; thus, the potential missing acetaldehyde precursors (from either a marine or terrestrial source) must have a longer lifetime.

As shown in Table 5, primary ocean emissions of acetaldehyde in the base simulation (22 Tg yr^−1^) are lower than previous work (57 Tg yr^−1^), likely due to updates to the model parameterization of the water transfer velocity (Johnson, 2010). Additional independent estimates of the ocean source are also much larger (34 to 42 Tg yr^−1^, Read et al., 2012; S. Wang et al., 2019). However, an increased primary ocean source would not address the bias in the free troposphere or in winter when biogenic activity from CDOM is zero in the model at high latitudes. Ship-borne measurements generally measure non-zero acetaldehyde seawater concentrations of approximately 5 nM (Read et al., 2012), and a recent trans-Atlantic campaign found that acetaldehyde concentrations from 47° S to 50° N did not always correlate with levels of CDOM (Yang et al., 2014). Therefore, we set a minimum seawater concentration of 5 nM in the model parameterization regardless of CDOM level. This change adds 2 Tg C yr^−1^ in emissions and increases concentrations over the remote ocean in winter by up to 50 ppt.

Figure 13 shows the combined effect of adding new ocean VOCs in Sect. 5 and improving the seawater parameterization described above on modeled acetaldehyde (labeled “Improve Ocean VOCs”). Although the direct ocean source in this work is lower than previous estimates as described above, the secondary source from precursor VOCs is enhanced. Of the additional marine VOCs described in Sect. 5, 19 Tg C yr^−1^ produce acetaldehyde as an oxidation product (Tables 3 and 4). This is compared to 12 Tg C yr^−1^ of direct emissions in the base model. These sources substantially increase average modeled acetaldehyde below 3 km, with the largest improvement during winter (40 to 60 ppt) when atmospheric lifetimes are longer and the influence of the ocean can extend aloft. In summer, the average model increase below 3 km is only 10 to 20 ppt due to higher OH concentrations. Recent work over North America suggested that free tropospheric VOCs may be underestimated due to errors in model vertical mixing (Chen et al. 2019), but in Northern Hemisphere summer slower mixing would not be expected to compensate for the short lifetime of acetaldehyde in this region (~ 5 h). Thus, the pervasive model bias in the free troposphere cannot be explained by an increase in known direct or indirect ocean sources.

Photodegradation of organic aerosols (OA) is another potential source of oxygenated VOCs such as acetaldehyde to the troposphere (Kwan et al., 2006; Epstein et al., 2014; Wong et al., 2015; S. Wang et al., 2019). The source of secondary organic aerosols (SOA) is uncertain and has been suggested to be up to 4 times larger than current estimates given an implied underestimate of the photochemical loss term (Hodzic et al., 2016). We test the potential impact of the maximum possible source of acetaldehyde from photochemical loss of OA by increasing the overall model production of SOA by a factor of 4 to maximize the impact of Reaction (R2) below. We apply a photolysis frequency for OA of 4 × 10^−4^*J*_NO2_ (Hodzic et al., 2015) to Reactions (R1) and (R2) as an upper limit and describe the formulation of Reactions (R1) and (R2) below.

(R1) OCPI+hv=0.5ALD2

(R2) SOAS+hv=0.66SOAS+ALD2

The model species OCPI and SOAS represent the majority of simulated OA in the remote atmosphere. OCPI is aged (hydrophilic) organic carbon (12 g C mol^−1^) and SOAS is SOA from all emissions categories (150 g mol^−1^). Both are assumed for the purposes of the sensitivity tests here to have an OA/OC ratio of 2.1. In Reaction (R1), one molecule of carbon (0.5 ALD2) is produced per reaction. In Reaction (R2), one acetaldehyde molecule (ALD2) is produced per reaction. The resulting impact on acetaldehyde is only appreciable in the Northern Hemisphere winter (Fig. 13), when modeled aerosol amounts are highest and the lifetime of acetaldehyde is long. Given that this test represents an upper limit, we conclude that photolysis of organic aerosols cannot provide a sufficient source of acetaldehyde to reconcile the model with observations.

We consider whether an entirely unknown VOC with moderate lifetime and a high yield of acetaldehyde at low NO_*x*_ could resolve the free tropospheric model bias. We emit such a species with a lifetime of approximately 1 month against oxidation by OH, emissions of 100 Tg yr^−1^ from either anthropogenic, biomass burning, or ocean sources, and a yield of one acetaldehyde molecule per reaction with OH. We do not test a terrestrial biogenic source here but expect the results would be similar to the biomass burning case. These simulations result in average tropospheric concentrations of 1 to 5 ppb. The effect of the unknown VOC is compatible with the model simulation of OH (unlike the addition of oceanic ALK4 needed to reconcile OHR observations as described in Sect. 5). The maximum cOHR_mod_ of this species is small (< 0.03 s^−1^). The impact on modeled acetaldehyde (Fig. 13) is generally similar across all three source categories due to the long lifetime of this precursor. As shown in Figs. 13 and 14, the addition of this unknown VOC modestly improves the simulation of acetaldehyde and PAA everywhere, but a large residual underestimate in Northern Hemisphere summer remains. The impact on PAN is minor, with the exception of Northern Hemisphere winter (Fig. 15), but this is likely driven by the model overestimate in NO_*y*_ (Fig. 7, Sect. 4.1).

Emission inventories of VOCs are known to be incomplete, for example neglecting emissions from volatile consumer products (McDonald et al., 2018) or failing to identify as much as half of emitted VOCs from biomass burning (Akagi et al. 2011), both of which peak in summer. The average emission factor for unidentified VOCs from biomass burning roughly corresponds to 75 Tg yr^−1^, similar to our sensitivity tests of 100 Tg yr^−1^ described above. However, recent attempts to quantify these unidentified VOCs (Stockwell et al., 2015; Koss et al., 2018) find that newly identified compounds tend to be too reactive to impact the remote atmosphere, as needed here; however, this work is ongoing and future efforts should investigate potential precursors of acetaldehyde that could be transported to the remote atmosphere. The missing source of precursor VOCs would need to have substantial additional summertime emissions above and beyond the sensitivity tests shown in Fig. 13 to address the Northern Hemisphere summertime bias. The required magnitude of this perturbation is difficult to reconcile within known measurement and emission uncertainty constraints.

## 6 Conclusions

The detailed set of chemical information available from the ATom field campaign provides the most comprehensive dataset ever collected to evaluate models in the remote atmosphere. The sampling strategy of collecting observations throughout the troposphere in multiple seasons is ideally suited for improving our understanding of tropospheric chemistry in a poorly observed region of the atmosphere. We use the first two deployments of the ATom field campaign during July–August 2016 and January–February 2017 to investigate sources of bias in model simulations of OH. Global models such as the GEOS-Chem CTM used here tend to overestimate the loss of methane by OH and underestimate CO, which provides the main tropospheric sink of OH. Comparisons of the model with observations from the first two ATom deployments do not show systematic bias in the simulation of OH or the drivers of remote OH production (water vapor, photolysis of ozone, ozone, and NO_*y*_), with the exception of wintertime NO_*y*_, which is overestimated by 70%.

The model overestimate of wintertime NO_*y*_ is largely attributable to nitric acid. This bias is not due to an anthropogenic inventory overestimate but may reflect insufficient wet scavenging as well as loss to sea-salt aerosols by nitric acid, although the former mechanism may be counteracted by photolysis of the resulting nitrate aerosols. The impact of resolving this wintertime NO_*y*_ bias is uncertain but could marginally reduce the model overestimate of OH. Future work should improve constraints on these mechanisms, which have all received only preliminary validation, and carefully examine their impact in the context of broader atmospheric chemistry, particularly NO_*y*_ partitioning throughout the troposphere.

We present the first comparison of measured OH reactivity (OHR) from aircraft with a global model to evaluate the tropospheric sink of OH. We calculate OH reactivity (cOHR_obs_) from relevant species observed during ATom and compare this to cOHR from the model (cOHR_mod_). Measured OHR is higher than cOHR_obs_ by approximately 0.2 to 0.4 s^−1^ below 3 km. This missing OHR correlates with acetaldehyde during summer, indicating a potential source of missing reactive VOCs, similar to the findings of Mao et al. (2009) and S. Wang et al. (2020). The addition of a comprehensive set of ocean emissions of VOCs increases global mean cOHR by 6% but cannot reproduce the observed OHR enhancement during ATom-1. Adding sufficient alkanes to the model to resolve this bias requires an improbably large ocean source (340 Tg C yr^−1^) and would degrade the model simulation of OH and NO_*y*_. Only one-third of the increase in cOHR in summer in this test is due to the alkanes; the rest is from oxidation products and changes in OH. Therefore, a more reactive VOC would still need to be emitted in large amounts.

The model successfully simulates the seasonality and hemispheric gradient in cOHR but has a persistent underestimate of up to 12% in the lowest 3 km, primarily due to missing model acetaldehyde. The model does not underestimate CO, with the exception of Northern Hemisphere winter, which has been previously recognized by Kopacz et al. (2010) and attributed to underestimated fossil fuel emissions. The inability to reproduce observations of remote acetaldehyde was first observed during the PEM-Tropics campaign (Singh et al., 2001, 2003; Millet et al., 2010), but the measurement was uncertain. Improvements in measurement precision and the accompanying measurement of PAA during ATom (S. Wang et al., 2019) strengthen the conclusion that there is a large amount of acetaldehyde present in the atmosphere that cannot be explained by current models. We investigate possible underestimates in known sources of acetaldehyde, including emissions of VOCs from anthropogenic, biomass, or oceanic sources or production from the photolysis of organic aerosols. No known source can fully resolve the bias in acetaldehyde throughout the troposphere, and particularly in the Northern Hemisphere summer. We consider the possibility that there is a large, diffuse source of unknown VOCs by implementing 100 Tg yr^−1^ of such a compound from ocean, biomass burning, or anthropogenic sources. This hypothetical source modestly reduces the model acetaldehyde bias and is compatible with the simulation of OH and cOHR; however, an additional source is required to resolve the largest bias in the Northern Hemisphere summer. Errors or omissions in the oxidation mechanism of known VOCs could be another source of bias. For example, significant uncertainties exist in peroxy radical (RO_2_) chemistry for large RO_2_ molecules (Praske et al., 2017), although the flux of carbon through a minor pathway would have to be large, restricting the possible known sources. Further laboratory and field observations are needed to understand which precursors and sources could lead to the sustained production of acetaldehyde observed during ATom and prior campaigns.

This study demonstrates that long-standing model biases in global mean OH are unlikely to be due to errors in simulating tropospheric chemistry over the ocean. This implies that a large bias must be present in OH production or loss over land and future work should focus on evaluating continental OH sources and sinks. Errors in modeled OH were recently investigated by Strode et al. (2015), and when overestimates related to production terms were corrected, model OH remained too high in the Northern Hemisphere, suggesting that future studies should focus on errors in OH loss.

## Supplementary Material

SI

## Figures and Tables

**Table 1.** Annual emissions of CO and NO_*x*_ for 2016 used in the GEOS-Chem simulations.

Emissions category	CO, Tg	Emissions category	NO_*x*_, Tg N
Fuel combustion*	590	Fuel combustion*	32.9
Biomass burning	311	Biomass burning	6.0
NMVOC oxidation	698	Soil emissions	7.8
Methane oxidation	936	Lightning emissions	6.0
Total	2535	Total	52.7

* Anthropogenic fossil fuel and biofuel combustion.

**Table 2.** Description of ATom measurements used to evaluate the model simulation.

Measurement	Instrument	Accuracy	Detection limit/precision	Reference
OHR	Airborne Tropospheric Hydrogen Oxides Sensor (ATHOS)	0.8 s^−1^	±0.4 s^−1^	Faloona et al. (2004); Mao et al. (2009)
Water vapor	Diode laser hygrometer (DLH)	5%	0.1% or 50 ppb	Diskin et al. (2002); Podolske et al. (2003)
NO_*y*_^1^	NOAA nitrogen oxides and ozone (NO_*y*_O_3_)		0.05 ppb^2^	Pollack etal. (2010); Ryerson et al. (1998, 2000)
Photolysis frequencies via actinic flux	Charged-coupled device Actinic flux Spectroradiometers (CAFS)	*j*O_3_ 20% *j*NO_2_ 12%	*j*O_3_ 10^−7^ s^−1^ *j* NO_2_ 10^−6^ s^−1^	Shetter and Mueller (1999), Petropavloskikh et al. (2007), Hofzumahaus et al. (2004)
Peroxyacetyl nitrate (PAN)	PAN and trace Hydrohalocarbon ExpeRiment (PANTHER)	10%	2 ppt ±10%	Elkins et al. (2001); Wofsy etal. (2011)
Components of OH reactivity^3^				
CH_4_	NOAA Picarro	0.6 ppb	0.3 ppb	Karion etal. (2013)
CO	Harvard Quantum Cascade Laser System (QCLS)	3.5 ppb	0.15 ppb	McManus et al. (2005); Santoni et al. (2014)
H_2_^4^	UAS Chromatograph for Atmospheric Trace Species (UCATS)/PANTHER		7.5 ppb^5^	Hintsa et al. (2020)
NO, NO_2_, O_3_	NOAA NO_*y*_O_3_		0.006, 0.03, 1.7 ppb^2^	Pollack etal. (2010); Ryerson et al. (1998, 2000)
Methyl hydroperoxide, nitric acid, hydrogen peroxide, peroxyacetic acid, peroxynitric acid	Caltech Chemical ionization mass spectrometer (CIMS)	±30%, ±30%, ±30%, ±50%, ±30%	25, 50, 50, 30, 100 ppt	St. Clair etal. (2010); Crounse et al. (2006)
Formaldehyde	NASA In Situ Airborne Formaldehyde (ISAF)	10%	10 ppt	Cazorla et al. (2015); DiGangi et al. (2011); Hottle et al. (2009)
Methanol, acetaldehyde, propane, dimethyl sulfide, ethanol, acetone, methyl ethyl ketone, propanal^6^, butanal^6^, toluene, methyl vinyl ketone, methacrolein i-Butane + n-butane + i-pentane + n-pentane^7^	NCAR Trace Organic Gas Analyzer (TOGA) 30%, 20%, 20%, 20%, 30%, 15%, 20%, 20% 15%, 15%, 15%, 15%	30%, 20%, 30%, 15%,	10, 10, 20, 2, 30, 10, 2, 20, 2, 0.6, 4, 2 2, 2, 4, 4 ppt	Apel et al. (2015)
OH, HO_2_	ATHOS	74% to 135%	0.018, 0.2 ppt	Faloona et al. (2004); Brune et al. (2020)
Ethane, benzene	UCI Whole air sampler (WAS)	5%, 5%	3, 3 ppt	Colman et al. (2001); Simpson et al. (2010)

1 Model NO_*y*_ is defined as NO + NO_2_ + HONO + HNO_3_ + HNO_4_ + 2 × N_2_O_5_ + ClNO_2_ + ΣPNs + ΣANs.

2 Average of 2*σ* uncertainty for each individual 1 Hz measurement for ATom-1 and ATom-2.

3 Included in cOHR are observations of species where at least 20% of the possible available measurements below 3 km are not missing.

4 The GEOS-Chem concentration of H_2_ is set to a constant value of 500 ppt.

5 Average of reported error for each individual measurement for ATom-1 and ATom-2.

6 Lumped as > C_4_ alkanes (ALK4) in GEOS-Chem.

7 Lumped as > C_3_ aldehydes (RCHO) in GEOS-Chem.

**Table 3.** Biogenic ocean emissions of VOCs.

GEOS-Chem 2 species^2^	No. of lumped species	Produces acetaldehyde?	Annual net emissions (Tg C)^1^	Reference for seawater concentration
ALD2	1	Yes	12.02	Millet et al. (2010)
MOH	1	No	−1.54	Personal communication, Dylan B. Millet, 2018
ACET	1	No	−75.65	Fischer et al. (2012)
LIMO	1	Yes	0.04	Hackenberg et al. (2017)
MTPA	3	Yes	0.05	Hackenberg et al. (2017)
MTPO	2	Yes	0.06	Hackenberg et al. (2017)
EOH	1	Yes	−5.52	Beale et al. (2010)
C2H6	1	Yes	0.33	Plass-Dülmer et al. (1993)
C2H4	1	No	0.75	Plass-Dülmer C. et al. (1993)
PRPE	2	Yes	0.95	Plass-Dülmer C. et al. (1993)
C3H8	1	Yes	0.16	Plass-Dülmer et al. (1993)
ALK4	2	Yes	0.12	Plass-Dülmer et al. (1993)
C2H2	1	No	0.02	Plass-Dülmer et al. (1993)
ISOP	1	Yes	1.64	Arnold et al. (2009)
RCHO	1	Yes	−1.03	Singh et al. (2003)
MEK	1	Yes	−7.214	Schlundt et al. (2017)
Total net emission			−74.82	
Total net emission producing acetaldehyde			1.60	

1 Net ocean emissions = upward flux out of the ocean–ocean uptake.

2 More information on the GEOS-Chem species definitions can be found here: http://wiki.seas.harvard.edu/geos-chem/index.php/Species_in_GEOS-Chem (last access: 21 May 2020).

**Table 4.** Abiotic ocean emissions of VOCs according to Brüggemann et al. (2018)^1^.

GEOS-Chem species^2^	No. of lumped species	Produces acetaldehyde?	Annual emission (Tg C)
ACET	1	No	10.07
EOH	1	Yes	5.16
ALD2	1	Yes	2.26
MOH		No	0.79
RCHO	21	Yes	3.88
ISOP	1	Yes	1.04
PRPE	13	Yes	4.44
MACR	1	Yes	0.42
ACTA	1	Yes	0.10
CH2O	1	No	0.03
XYLE	1	No	0.05
TOLU	1	No	0.04
BENZ	1	No	0.02
Total net emission			28.30
Total net emission producing acetaldehyde			17.30

1 Table S2 shows the emission factor assumed for each species and the lumping methodology for Table 4.

2 More information on the GEOS-Chem species definitions can be found here: http://wiki.seas.harvard.edu/geos-chem/index.php/Species_in_GEOS-Chem (last access: 21 May 2020).

**Table 5.** Model sources of acetaldehyde in 2016.

Sources (Tg yr^−1^)*	Millet et al. (2010)	This work
Photochemical production	128	166
Net ocean emission	57	22
Terrestrial plant growth + decay	23	26
Biomass burning	3	3
Anthropogenic emission	2	2
Total source	213	219

* Emissions are given in Tg of acetaldehyde per year for comparison to Millet et al. (2010). These totals are for the baseline model simulation described in Sect. 2.1.
