# Supplementary material for: Constraining remote oxidation capacity with ATom observations
Source: Atmos Chem Phys. Author manuscript; Available in PMC 2021 Mar 8. (PMC7939060; doi:10.5194/acp-20-7753-2020)
Supplement: SI [file NIHMS1669560-supplement-SI.pdf]

Supplement of Atmos. Chem. Phys., 20, 7753–7781, 2020
https://doi.org/10.5194/acp-20-7753-2020-supplement
© Author(s) 2020. This work is distributed under
the Creative Commons Attribution 4.0 License.

Atmospheric Chemistry and Physics
Open Access

Supplement of

Constraining remote oxidation capacity with ATom observations

Katherine R. Travis et al.

Correspondence to: Katherine R. Travis (katherine.travis@nasa.gov) and Colette L. Heald (heald@mit.edu)

The copyright of individual parts of the supplement might differ from the CC BY 4.0 License.

Supplemental Information

Section 1. Description of chemistry added to GEOS-Chem for unsaturated C_2 compounds and organic acids.

Chemistry added to GEOS-Chem.

Table S1.

Species added

```
C2H2      = IGNORE; {C2H2; Acetylene}
C2H4      = IGNORE; {C2H4; Ethene}
EO2       = IGNORE; {HOCH2CH2O2; Peroxy radical from C2H4}
EO        = IGNORE; {HOCH2CH2O; from C2H4}
```

Chemistry added

```
C2H2 + OH = GLYX + OH :                                 GCKMT17(0.636);
C2H2 + OH = HCOOH + CO + HO2 :                          GCKMT17(0.364);
C2H4 + OH = 0.750EO2 + 0.500CH2O + 0.250HO2 :           GCKMT15();
EO2 + NO = EO + NO2:                                    GCARR(4.2E-12,0.0E+00,180.0);
EO + O2 = GLYC + HO2:                                   1.00E-14;
C2H4 + O3 = CH2O + 0.120HO2 + 0.500CO +                 GCARR(1.2E-14,0.0E+00, -2630.);
        0.120OH + 0.500HCOOH:                                     {Lamarque et al., 2012}
```

In GEOS-Chem, RCOOH, or organic acids produced during VOC oxidation, do not themselves undergo further oxidation and thus are a loss of carbon in the model. We include oxidation of RCOOH parameterized as propionic acid from the MCMv3.3.1.

Table S2.

Species added

```
RCOOH     = IGNORE; {C2H5C(O)OH; > C2 organic acids}
RCO2      = IGNORE; {Peroxy from RCOOH}
```

Chemistry added

```
RCOOH + OH = RCO2 :                   GCARR(1.2E-12, 0.0E+00, 0.0) ;
RCO2 + HO2 = ETP :                    GCARR(4.3E-12, 0.0E+00, 870.0) ;
RCO2 + NO = ALD2 + HO2 + NO2 :        GCARR(2.55E-12, 0.0E+00, 380.0) ;
```

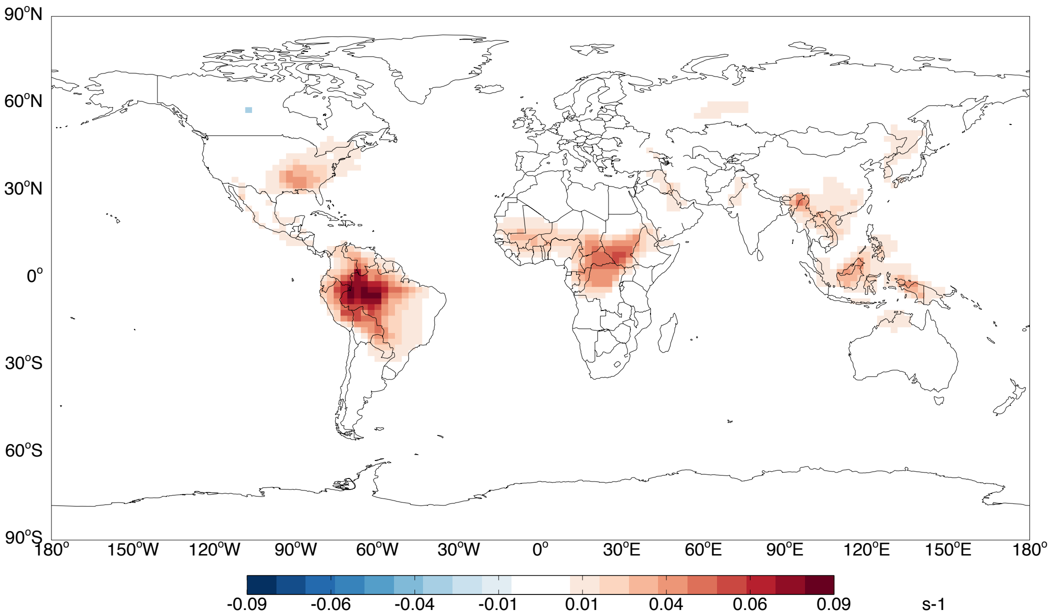

Figure S1. One-month test of the impact of RCOOH on surface cOHR, including the chemistry from Table S2.

Section 2. Model variables included in the calculation of Figure. 1

Table S3. Variables included in the calculated OH reactivity shown in Figure 1.

GEOS-Chem Species[1]	Species description
ACET	Acetone
ACTA	Acetic acid
ALD2	Acetaldehyde
ALK4	>C_4 alkanes
BENZ	Benzene
Br2	Molecular bromine
BrO	Bromine monoxide
C2H2	Ethyne (Acetylene)
C2H4	Ethylene
C2H6	Ethane
C3H8	Propane
CH2Br2	Dibromomethane
CH2Cl2	Dichloromethane
CH2O	Formaldehyde
CH3Br	Methyl bromide
CH3Cl	Methyl chloride
CH3I	Methyl iodide
CH4	Methane
CHBr3	Bromoform
CHCl3	Chloroform

GEOS-Chem Species[1]	Species description
Cl2	Molecular chlorine
ClNO2	Nitryl chloride
ClNO3	Chlorine nitrate
ClO	Chlorine monoxide
CO	Carbon monoxide
DMS	Dimethyl sulfide
EOH	Ethanol
ETHLN	Ethanal nitrate
GLYC	Glycoaldehyde
GLYX	Glyoxal
H2	Molecular hydrogen
H2O2	Hydrogen peroxide
HAC	Hydroxyacetone
HBr	Hydrobromic acid
HC187	Epoxide oxidation product m/z 187-189
HCl	Hydrochloric acid
HCOOH	Formic acid
HI	Hydrogen iodide
HNO2	Nitrous acid
HNO3	Nitric acid
HNO4	Peroxynitric acid
HO2	Hydroperoxy radical
HOCl	Hypochlorous acid
HOI	Hypoiodous acid
HONIT	2nd generation monoterpene organic nitrate
HPALD	Hydroperoxyaldehydes
I2	Molecular iodine
IEPOXA	trans-β isoprene epoxydiol
IEPOXB	cis-β isoprene epoxydiol
IEPOXD	δ isoprene epoxydiol
IMAE	C_4 epoxide from oxidation of PMN
IPMN	Peroxymethacryloyl nitrate (MPAN) from isoprene oxidation
ISN1	Nighttime isoprene nitrate
ISOP	Isoprene
ISOPNB	Isoprene nitrate Beta
ISOPND	Isoprene nitrate Delta
LIMO	Limonene
LVOC	Gas-phase low-volatility non-IEPOX product of ISOPOOH (RIP) oxidation

GEOS-Chem Species[1]	Species description
MACR	Methacrolein
MACRN	Nitrate from MACR
MAP	Peroxyacetic acid
MEK	Methyl ethyl ketone
MGLY	Methylglyoxal
MOBA	5C acid from isoprene
MOH	Methanol
MONITS	Saturated 1st generation monoterpene organic nitrate
MONITU	Unsaturated 1st generation monoterpene organic nitrate
MHP	Methylhydroperoxide
MTPA	Lumped monoterpenes (α-pinene, β-pinene, sabinene, carene)
MTPO	Terpinene, terpinolene, myrcene, ocimene, other monoterpenes
MVK	Methyl vinyl ketone
MVKN	Nitrate from MVK
NO	Nitric oxide
NO2	Nitrogen dioxide
NO3	Nitrate radical
NPMN	Non-isoprene peroxymethacryloyl nitrate (MPAN)
O3	Ozone
OClO	Chlorine dioxide
OH	Hydroxyl radical
PROPNN	Propanone nitrate
PRPE	$\geq C_3$ alkenes
R4N2	$\geq C_4$ alkylnitrates
RCHO	$\geq C_3$ aldehydes
RCOOH	$>C_2$ organic acids
RIPA	1,2-ISOPOOH (Peroxide from RIO2)
RIPB	4,3-ISOPOOH (Peroxide from RIO2)
RIPD	δ(1,4 and 4,1)-ISOPOOH (Peroxide from RIO2)
SO2	Sulfur dioxide
TOLU	Toluene
XYLE	Xylene

[1] For more information, visit http://wiki.seas.harvard.edu/geos-chem/index.php/Species_in_GEOS-Chem

Section 3. Distributions of observed and modeled OH.

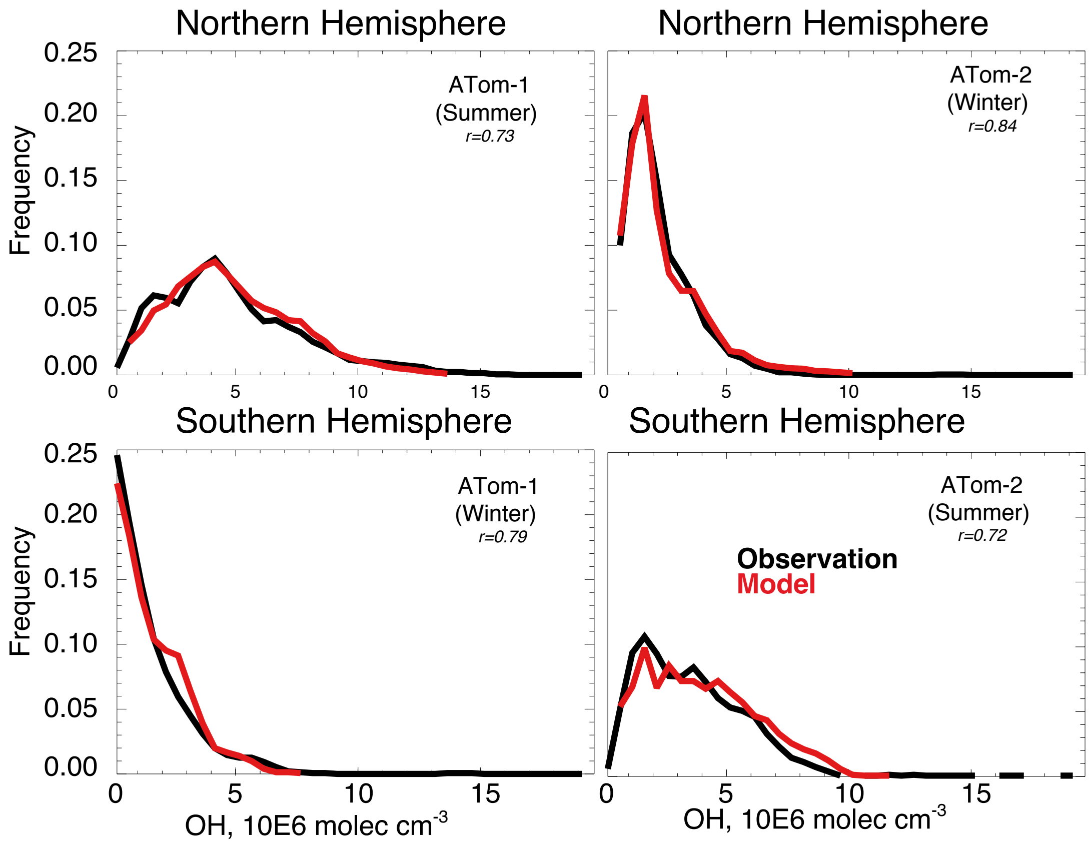

Figure S2. Frequency distributions of OH from the model and observations filtered as described in Figure 3.

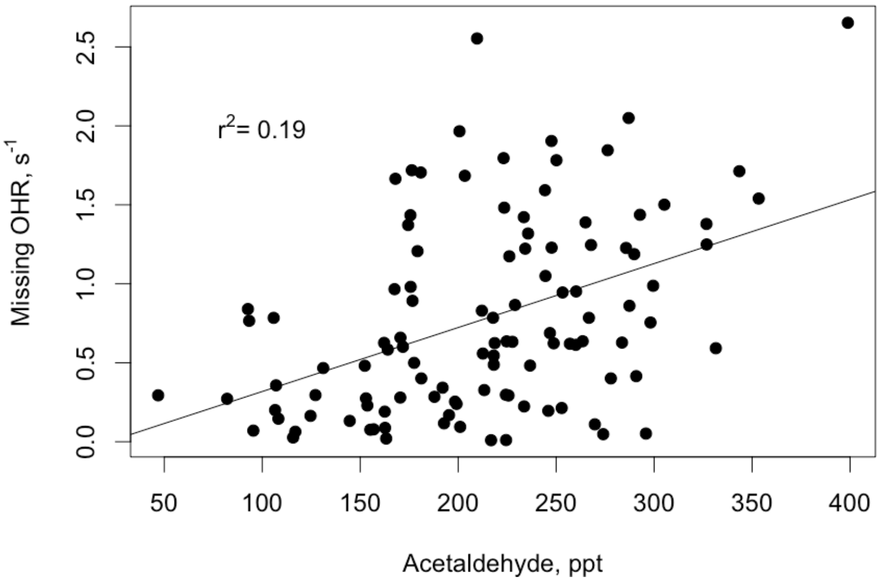

Figure S3. Observations of acetaldehyde and the difference between OHR and $cOHR_{obs}$ below 3 km in the Northern Hemisphere during ATom-1. The data has been filtered to remove biomass burning (acetonitrile >200 ppt) influence.

Section 4. Emission factors used to generate Table 4.

Table S4. Emission factor assumed for each species and the lumping methodology for Table 4.

PTR-TOF-MS	# carbons	Species Identified	Assumed species	GEOS-Chem species	EF[1], production / 10^7 molec. mW^{-1} s^{-1}	EF*1E[7]*Carbons*12gC $mmol^{-1}$	EF*1E[7]* molecular weight	Matlab Tool (Tg/yr)
42.034	2	C2H3N (acetonitrile)	Acetonitrile					27.1318
59.047	3	C3H6O (acetone or propanal?)	Acetone	ACET	77.391	2.79E+10	4.49E+10	16.3006
47.048	2	C2H6O (ethanol)	Ethanol	EOH	59.672	1.43E+10	2.75E+10	10.0142
45.032	2	C2H4O (acetaldehyde)	Acetaldehyde	ALD2	26.127	6.27E+09	1.15E+10	4.1974
33.034	1	CH4O (methanol)	Methanol	MOH	16.375	1.97E+09	5.25E+09	1.9302
73.061	4	C4H8O (butanal)	2-methylpropanal	RCHO	4.63	2.22E+09	3.34E+09	1.2067
69.067	5	C5H8 (isoprene)	Isoprene	ISOP	4.788	2.87E+09	3.26E+09	1.1801
57.032	3	C3H4O (acrolein)	Acrolein	RCHO	5.355	1.93E+09	3.00E+09	1.0904
43.054	3	C3H6	Propene	PRPE	6.593	2.37E+09	2.77E+09	1.0121
57.067	4	C4H8 (butene)	Butene	PRPE	4.058	1.95E+09	2.28E+09	0.8265
83.08	6	C6H10 (cyclohexene)	Cyclohexene	PRPE	2.317	1.67E+09	1.90E+09	0.6875
71.045	4	C4H6O (methacrolein)	Methacrolein	MACR	2.397	1.15E+09	1.68E+09	0.6082
127.101	8	C8H14O	2-octenal	PRPE	1.178	1.13E+09	1.49E+09	0.535
85.059	5	C5H8O	2-Methylbut-3-yn-2-ol	RCHO	1.575	9.45E+08	1.32E+09	0.4794
87.075	5	C5H10O	Pentanal	RCHO	1.46	8.76E+08	1.26E+09	0.4535
70.061	4	C4H7N	Butyronitrile	NA	1.7	8.16E+08	1.17E+09	0.4249
41.038	3	C3H4	Cyclopropene	PRPE	2.761	9.94E+08	1.11E+09	0.404
111.104	8	C8H14	Cyclooctene	PRPE	1.01	9.70E+08	1.11E+09	0.4003
97.094	7	C7H12	Cycloheptene	PRPE	1.023	8.59E+08	9.83E+08	0.3533
51.043	1	CH6O2	Methanol-hydrate	MOH	1.895	2.27E+08	9.48E+08	0.3459
113.088	7	C7H12O	2-heptenal	RCHO	0.796	6.69E+08	8.92E+08	0.3227

PTR-TOF-MS	# carbons	Species Identified	Assumed species	GEOS-Chem species	EF[1], production / 10^7 molec. mW^{-1} s^{-1}	EF*1E[7]*Carbons*12gC $mmol^{-1}$	EF*1E[7]* molecular weight	Matlab Tool (Tg/yr)
43.017	2	C2H2O (ketene)	NA	NA?	1.904	4.57E+08	8.00E+08	0.2916
71.077	5	C5H10	1-pentene	PRPE	1.041	6.25E+08	7.30E+08	0.2637
129.116	8	C8H16O	3-octanone	RCHO	0.545	5.23E+08	6.98E+08	0.2533
61.026	2	C2H4O2	Acetic acid	ACTA	1.134	2.72E+08	6.81E+08	0.246
109.093	8	C8H12	1,5-cyclooctadiene	PRPE	0.546	5.24E+08	5.90E+08	0.214
143.128	9	C9H18O	5-nonanone	RCHO	0.398	4.30E+08	5.66E+08	0.2042
101.088	6	C6H12O	2-methylpentanal	RCHO	0.564	4.06E+08	5.64E+08	0.2019
63.041	2	C2H6O2	Ethylene glycol	RCHO	0.884	2.12E+08	5.48E+08	0.1979
107.042	7	C7H6O	Benzaldehyde	RCHO	0.514	4.32E+08	5.45E+08	0.1947
121.056	8	C8H8O	2-methylbenzaldehyde	RCHO	0.431	4.14E+08	5.17E+08	0.1857
87.039	4	C4H6O2	1,4-dihydroxy-2-butyne	RCHO	0.593	2.85E+08	5.10E+08	0.1832
141.112	9	C9H16O	trans-2-nonenal	RCHO	0.359	3.88E+08	5.03E+08	0.1812
95.079	7	C7H10	Cyclopentylacetylene	PRPE	0.532	4.47E+08	5.01E+08	0.1798
111.072	7	C7H10O	Dicyclopropyl methanone	RCHO	0.445	3.74E+08	4.90E+08	0.1783
101.052	5	C5H8O2	Acetylacetone	RCHO	0.461	2.77E+08	4.61E+08	0.1658
139.108	9	C9H14O	Isophorone	RCHO	0.332	3.59E+08	4.59E+08	0.1638
153.114	1	C10H16O	α-pinene oxide	RCHO	0.281	3.37E+07	4.27E+08	0.1529
99.074	6	C6H10O	2-hexenal	RCHO	0.405	2.92E+08	3.97E+08	0.1449
123.106	9	C9H14	Cyclohexylallene	PRPE	0.311	3.36E+08	3.80E+08	0.1361
77.055	3	C3H8O2	2-methoxyethanol	RCHO	0.445	1.60E+08	3.38E+08	0.1237
81.065	6	C6H8	1,3-cyclohexadiene	PRPE	0.355	2.56E+08	2.84E+08	0.1041
125.087	8	C8H12O	4-Acetylcyclohexene	RCHO	0.218	2.09E+08	2.71E+08	0.0982
84.082	5	C5H9N	Pentanenitrile	NA	0.271	1.63E+08	2.25E+08	0.081
115.102	7	C7H14O	2-heptanone	RCHO	0.191	1.60E+08	2.18E+08	0.078

PTR-TOF-MS	# carbons	Species Identified	Assumed species	GEOS-Chem species	EF[1], production / 10^7 molec. mW^{-1} s^{-1}	EF*1E^7*Carbons*12gC $mmol^{-1}$	EF*1E^7* molecular weight	Matlab Tool (Tg/yr)
31.019	1	CH2O (formaldehyde)	Formaldehyde	CH2O	0.704	8.45E+07	2.11E+08	0.0775
67.051	5	C5H6 (cyclopentadiene)	1,3-cyclopentadiene	PRPE	0.291	1.75E+08	1.92E+08	0.0694
121.085	5	C5H12O3	2-(hydroxymethyl)-2-methyl-1,3-propanediol	NA	0.154	9.24E+07	1.85E+08	0.0648
68.048	4	C4H5N	pyrrole	NA	0.247	1.19E+08	1.66E+08	0.0607
58.036		NA	NA	NA	0.289	0.00E+00	1.65E+08	0.06
107.077	8	C8H10	Xylenes	XYLE	0.149	1.43E+08	1.58E+08	0.0573
72.046	3	C3H5NO	NA	NA	0.206	7.42E+07	1.46E+08	0.054
56.054	3	C3H5N	NA	NA	0.226	8.14E+07	1.24E+08	0.046
83.044	5	C5H6O	NA	NA	0.15	9.00E+07	1.23E+08	0.0444
49.01	1	CH4S	NA	NA	0.235	2.82E+07	1.13E+08	0.042
60.079	3	C3H9N	NA	NA	0.187	6.73E+07	1.10E+08	0.0407
93.063	7	C7H8	Toluene	TOLU	0.116	9.74E+07	1.07E+08	0.0398
151.099	1	C10H14O	NA	NA	0.07	8.40E+06	1.05E+08	0.0377
141.079	8	C8H12O2	NA	NA	0.067	6.43E+07	9.39E+07	0.0352
73.024	3	C3H4O2	NA	NA	0.125	4.50E+07	9.00E+07	0.0339
135.075	9	C9H10O	NA	NA	0.067	7.24E+07	8.98E+07	0.0337
97.059	6	C6H8O	NA	NA	0.092	6.62E+07	8.84E+07	0.0312
79.05	6	C6H6 (benzene)	Benzene	BENZ	0.092	6.62E+07	7.18E+07	0.0254
44.022	2	C2H3O	NA	NA	0.136	3.26E+07	5.85E+07	0.022
108.043	6	C6H5NO	NA	NA	0.045	3.24E+07	4.82E+07	0.0193
58.065	3	C3H7N	NA	NA	0.084	3.02E+07	4.79E+07	0.0166
137.084	9	C9H12O	NA	NA	0.017	1.84E+07	2.31E+07	0.0098

[1]Emission factor from (Brüggemann et al., 2017) Table S2 for biofilms on day 6.

Section 5. Incremental impact of additional ocean emissions over the baseline model simulation.

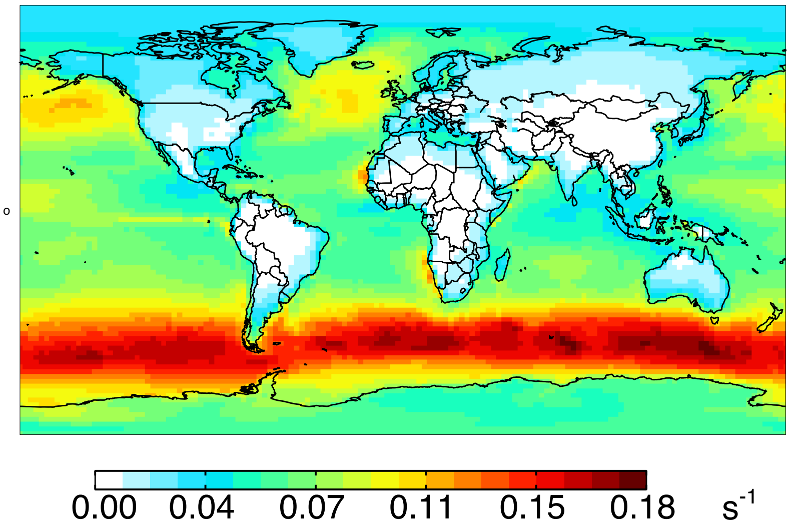

Figure S4. Impact of additional ocean emissions (Tables 3 and 4) over the baseline model (which includes methanol, acetone, and acetaldehyde) on annual simulated 2016 surface cOHRas described in the text.

Section 6. Description of MHP Interference

Recent laboratory work has shown methanediol (HOCH2OH, hydrated formaldehyde) is detected efficiently in the Caltech CIMS instruments at the same signals used to quantify MHP. Under high water vapor mixing ratios, as found in the lower atmosphere, HOCH2OH is likely detected in the Caltech CIMS with substantially greater efficiency than MHP on a molar basis, thus potentially amplifying the interference. Work is ongoing to better understand and quantify this issue.

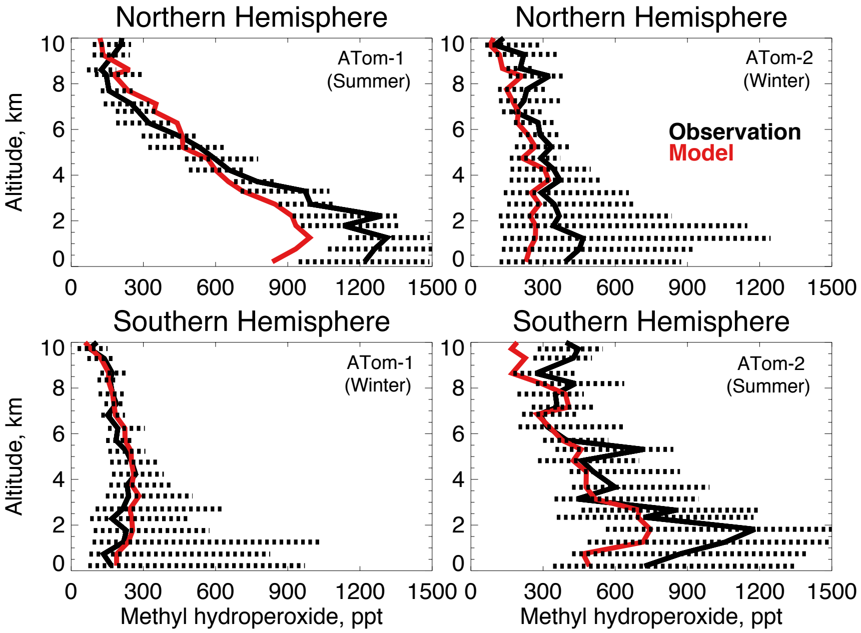

Figure S5. The same as Figure 3 for median methyl hydroperoxide profiles. Methyl hydroperoxide is measured by the Caltech CIMS instrument as described in Table 2.

Section 7. Zonal average plots of acetaldehyde for each deployment.

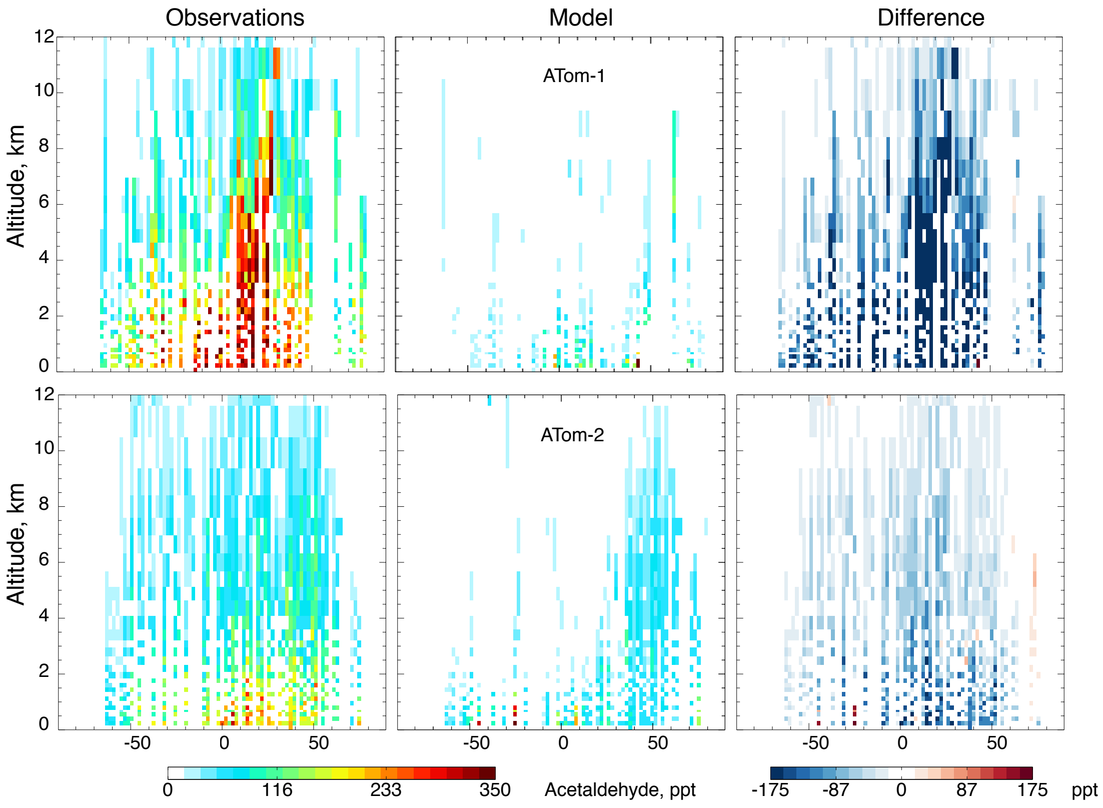

Figure S6. Zonal mean acetaldehyde gridded to the model resolution along the flight tracks over the ocean. Acetaldehyde is measured by the TOGA instrument as described in Table 2.

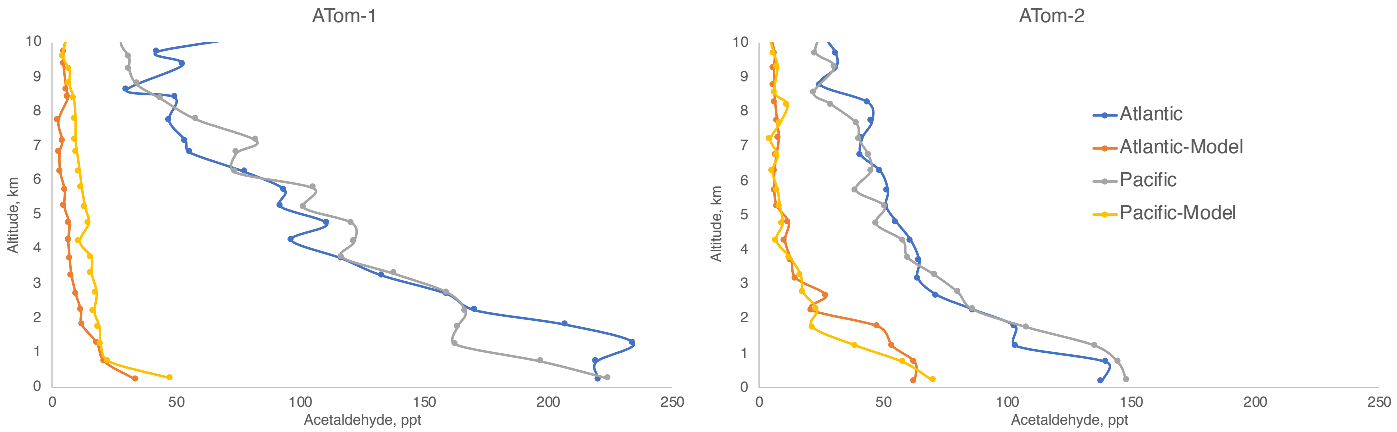

Figure S7. Acetaldehyde comparison split at 70ºW to represent the Pacific and Atlantic oceans, respectively. Acetaldehyde is measured by the TOGA instrument as described in Table 2.

Section 8. Model comparison with observed ethane and propane.

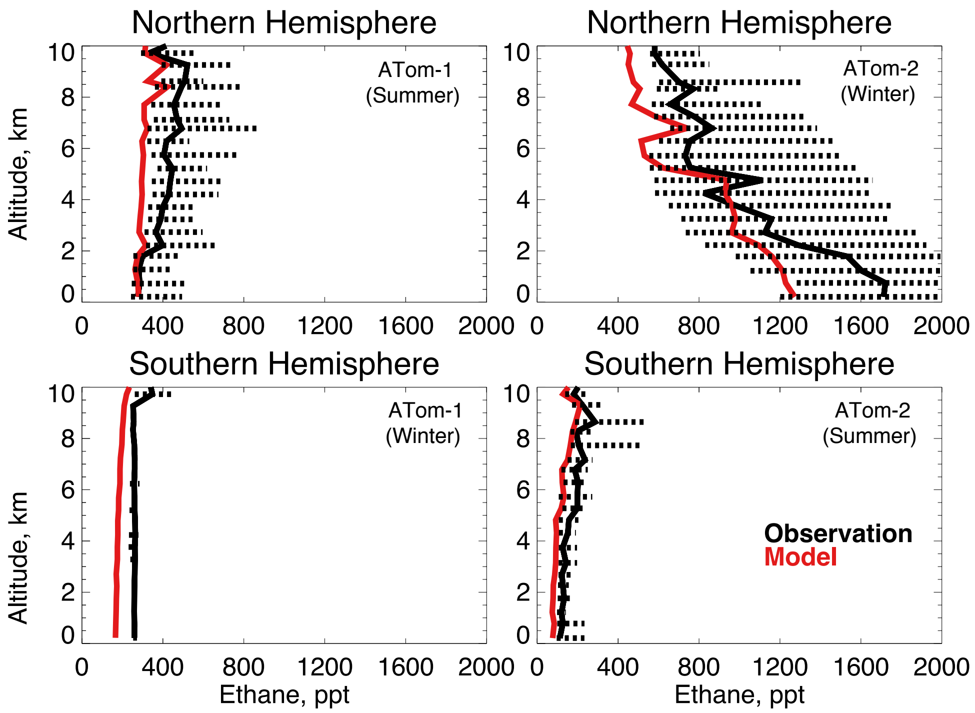

Figure S8. The same as Figure 3 for median ethane profiles. Ethane is measured by the UCI Whole Air Sampler (WAS) instrument as described in Table 2.

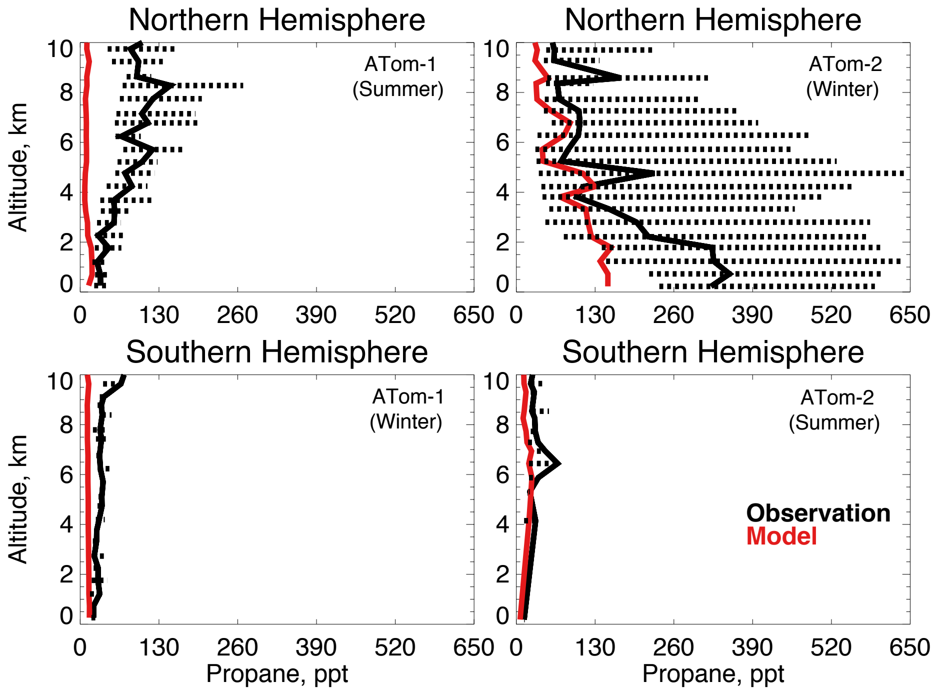

Figure S8. The same as Figure 3 for median propane profiles. Propane is measured by the Trace Organic Gas Analyzer (TOGA) instrument as described in Table 2.

Table S5. GEOS-Chem Ethane and Propane Chemistry (Adapted from Safieddine et al., 2017)

**Reaction**	**Rate**
**C2H6**	
C2H6 +OH→ETO2 +H2O	7.66E-12 exp(-1020/T)
C2H6 +NO3→ETO2 +HNO3	1.4E-18
ETO2 +NO→ALD2 +NO2 +HO2	2.6E-12 exp(365/T)
ETO2 + HO2→ETP	7.4E-13 exp(700/T)
ETO2 + MO2 →0.75CH2O +0.75ALD2 +HO2 +0.25MOH +0.25EOH	3E-13
ETO2 +ETO2→2ALD2 +2HO2	4.1E-14
ETO2 +ETO2→EOH +ALD2	2.7E-14
ETO2 + MCO3→MO2 +ALD2 +HO2 +{CO2}	1.68E-12 exp(500/T)
ETO2 + MCO3→ACTA +ALD2	1.87E-13 exp(500/T)
ETP+ hν→ OH+ HO2+ ALD2	
ETP +OH→0.64OH +0.36ETO2 +0.64ALD2	5.18E-12 exp(200/T)
**C3H8**	
C3H8 +OH→B3O2	k1=7.6E-12 exp(-585/T); k2=5.87*(300/T)$^{0.64}$ exp(- 816/T); K=k1 / (1+k2)
C3H8 +OH→A3O2	k1=7.60E-12 exp(-585/T); k2= 0.17*(300/T)$^{-0.64}$ exp(816/T) K=k1 / (1+k2)
A3O2 +NO→NO2 +HO2 +RCHO	2.9E-12 exp(350/T)
A3O2 +HO2→RA3P	2.91E-13exp(1300/T)[1-exp(-0.245*n)], n=3
A3O2 +MO2→HO2 +0.75CH2O +0.75RCHO +0.25MOH+0.25ROH	5.92E-13
A3O2+ MCO3 →MO2 +RCHO +HO2 +{CO2}	1.68E-12 exp(500/T)
A3O2 + MCO3 →ACTA +RCHO	1.87E-13 exp(500/T)
B3O2 +NO→NO2 +HO2 +ACET	2.7E-12 exp(350/T)
B3O2 +HO2→RB3P	2.91E-13exp(1300/T)[1-exp(-0.245*n)],n=3
B3O2 +MO2→0.5HO2 +0.5ACET +0.25ACET+0.75CH2O+0.25MOH +0.25ROH +0.5HO2 +0.021{CO2}	8.37E-14
B3O2 +MCO3→MO2 +HO2 +ACET +{CO2}	1.68E-12 exp(500/T)
B3O2 +MCO3→ACET +ACTA	1.87E-13 exp(500/T)
RA3P +OH→0.64OH +0.36A3O2 +0.64RCHO	5.18E-12 exp(200/T)
RB3P +OH→0.791OH +0.209B3O2 +0.791ACET	8.78E-12 exp(200/T)
RA3P+ hν→ OH+ HO2+ RCHO	
RB3P+ hν→ OH+ HO2+ ACET	
